# Extension of the 5-alkynyluridine side chain via C–C-bond formation in modified organometallic nucleosides using the Nicholas reaction

**DOI:** 10.3762/bjoc.16.1

**Published:** 2020-01-02

**Authors:** Renata Kaczmarek, Dariusz Korczyński, James R Green, Roman Dembinski

**Affiliations:** 1 Department of Bioorganic Chemistry, Centre of Molecular and Macromolecular Studies, Polish Academy of Sciences, Sienkiewicza 112, 90-363 Łódź, Poland; 2 Department of Chemistry and Biochemistry, University of Windsor, Windsor, Ontario, N9B 3P4, Canada,; 3 Department of Chemistry, Oakland University, 146 Library Drive, Rochester, Michigan 48309-4479, USA

**Keywords:** alkynes, 5-alkynyluridines, C–C-bond formation, dicobalt hexacarbonyl complexes, Nicholas reaction, nucleosides, propargyl cation

## Abstract

Dicobalt hexacarbonyl nucleoside complexes of propargyl ether or esters of 5-substituted uridines react with diverse C-nucleophiles. Synthetic outcomes confirmed that the Nicholas reaction can be carried out in a nucleoside presence, leading to a divergent synthesis of novel metallo-nucleosides enriched with alkene, arene, arylketo, and heterocyclic functions, in the deoxy and ribo series.

## Introduction

Nucleoside analogs are molecules of high pharmacological interest for the treatment of various conditions, especially cancer and viral diseases [1–5]. The substitution at C-5 of the uracil nucleobase provides a common framework for materials with potent biological properties [6–10]. Modification on this site of the nucleobase usually does not interfere with Watson–Crick base pairing. For example, C-5-modified pyrimidines are well tolerated by commercial polymerases [11–12]. Alkynyl modifications not only provide a biological impact but also create a synthetic handle for further functionalization/modification. Among others, alkynyl uridines undergo cycloisomerization to potent antiviral agents, furopyrimidines [13], related halofuropyrimidines [14], and can be converted into interstrand dimers [15].

In parallel, bioorganometallic chemistry provides new tools to influence biological interactions [16–24]. Cobalt possesses a diverse array of properties that can be manipulated to yield promising drug candidates [25]. The antiproliferative properties [26], as well as carbon monoxide-releasing properties [27–28] of dicobalt hexacarbonyl alkyne complexes have been noted, and their medicinal potential has been summarized [29–31].

Despite developments, the collection of metallo-nucleosides is limited. Hybridization of alkyl and aryl-substituted alkyne cobalt hexacarbonyls with 2'-deoxyuridines revealed pronounced in vitro activity against MCF-7 and MDA-MB-231 human breast cancer cells [32–33]. A recent investigation of hexacarbonyl dicobalt adducts of nucleosides containing derivatives of propargyl alcohol demonstrated their antiproliferative activities for the HeLa and K562 cell lines [31]. The formation of a reactive oxygen species in the presence of cobalt compounds was determined in K562 cells. The results indicate that the mechanism of action for most antiproliferative cobalt compounds may be related to the induction of oxidative stress [31]. Consequently, we aimed to develop methods that would synthetically extend the design of the metallo-nucleosides by introducing functionalized ligands in divergent synthesis. We decided to pursue the Nicholas reaction in the presence of the labile nucleoside unit, further modifying the already available material containing the propargyl alcohol derivative unit.

The chemistry of cationic propargyl dicobalt complexes, recognized as the Nicholas reaction, has become one of the most widely appreciated forms of metalorganic chemistry. These cations are generated most commonly from propargyl alcohol, -ether, or -acetate hexacarbonyl dicobalt complexes and a Lewis or Brønsted acid. A range of heteroatom nucleophiles have been incorporated into alkyne dicobalt complexes by this chemistry [34–40]. However, reactions with carbon-based nucleophiles provide an opportunity to access the structurally diverse products via formation of C–C bonds. Nucleophiles as diverse as electron-rich arenes or heteroarenes [41–42], alkenes [43], allylmetalloids [44–46], enol derivatives [47–48], and organometallics [49] are suitable for the Nicholas reaction. Allenic byproducts are rarely seen, and intramolecular versions of the reaction are also highly successful [50–51].

Although the Nicholas reaction has been employed to functionalize biomolecules, including amino acids [52–53], β-lactams [54], steroids [55], and carbohydrates [56–62], we are unaware of any examples of nucleoside functionalization by way of propargyl dicobalt cation chemistry. Nucleoside modifications are considerably challenging due to the presence of reactive functional groups. Since numerous uridine C-5 modifications play an important role in biochemistry, we considered exploration of pertinent methods development warranted, which at the same time may provide biologically active compounds.

## Results and Discussion

**Scheme 1:**
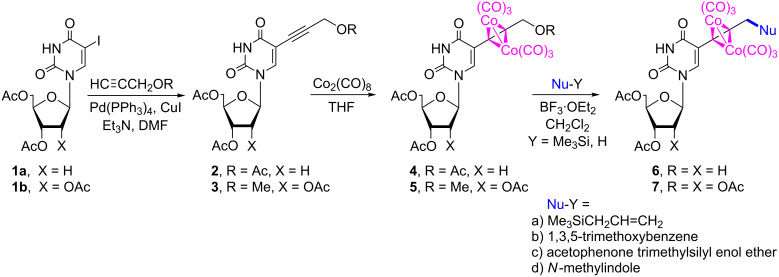
Preparation of (2'-deoxy)-5-alkynyluridines **2** and **3**, their dicobalt hexacarbonyl derivatives **4** and **5**, and the subsequent Nicholas reaction.

Preparation of 5-alkynyluridines was carried out from acyl-protected 5-iodouridines (**1a**,**b**) [8,63] and the appropriate terminal alkyne in the presence of catalytic amounts of Pd(PPh_3_)_4_, copper(I) iodide, triethylamine, in DMF, and at room temperature – to avoid cycloisomerization to furopyrimidines (Scheme 1). The modified pyrimidine nucleoside scaffolds, propargyl acetate-substituted 2'-deoxyuridine (R = Ac, **2**) and propargyl methyl ether-substituted uridine (R = Me, **3**), were obtained in 87% and 61% yield, respectively. These specific combinations were not optimized since we presumed that acetate and methyl ether could be used interchangeably. Acetyl protection has been introduced to alcohol functions to prevent free hydroxy groups from competing with the C-nucleophiles in the Nicholas reaction. The structures of alkynyl nucleosides **2** and **3** were confirmed by ^1^H and ^13^C NMR spectroscopy and high-resolution mass spectrometry [64–65].

The conversion of alkynyl nucleosides **2** and **3** into the corresponding dicobalt hexacarbonyl nucleosides complexes of **4** and **5** was accomplished at room temperature (Co_2_(CO)_8_, THF, 22 °C, 1 h) with 88–77% yield after silica gel column chromatography (Scheme 1). The structures of nucleosides **4** and **5** were confirmed by NMR and IR spectroscopy (for the synthesis of a related unprotected nucleoside, see [31]). The MS spectra of **4** and **5** exhibited appropriate high resolution molecular ions’ masses.

**Figure 1:**
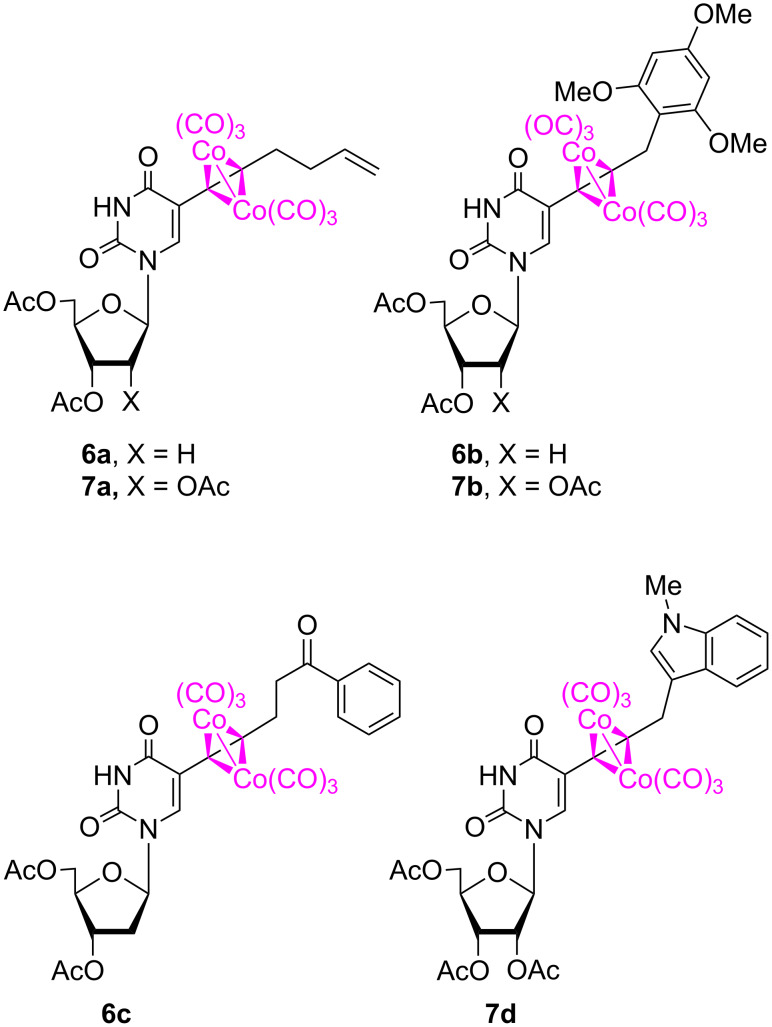
Structures of nucleosides **6** and **7**, products of the Nicholas reaction.

The solutions of uridine complexes **4** and **5** in dichloromethane were subjected to Nicholas reactions with a variety of diverse nucleophiles in the presence of BF_3_·OEt_2_. Representatives of the major classes of C-based nucleophiles in Nicholas reaction chemistry were selected, including electron-rich arenes, π-excessive heterocycles, enol derivatives, and allylmetalloids. Specifically, the reactivity of 1,3,5-trimethoxybenzene, *N*-methylindole, acetophenone trimethylsilyl enol ether, and allyltrimethylsilane was investigated (Table 1). The Nicholas reaction products **6** and **7** (Figure 1) were obtained successfully in moderate to good yields (Table 1). The reactions progressed quite slowly and required an excess amount of the Lewis acid (4–5 equiv) to proceed at a preparatively reasonable rate (Table 1, entries 1–4). These observations can be attributed to the substantial number of potentially competing Lewis basic sites in **4** and **5**. The use of tin(IV) chloride (stannic chloride) provided generally a slightly faster reaction but with slightly lower yields, except in the case of the **5**/allyltrimethylsilane/**7a** combination (Table 1, entry 5). Reactions were more successful when the amount of nucleophile present was in slight excess relative to that of the Lewis acid, whereas limited amounts of nucleophile resulted in greater amounts of decomposition. Slightly more decomposition products were observed by TLC in reactions with ribo nucleoside **5** (Table 1, entries 4–7) than with 2'-deoxy derivative **4** (Table 1, entries 1–3), leading to higher yields for nucleosides **6a**–**c** relative to **7a**,**b**,**d** (Table 1).

**Table 1:** Preparation of modified uridine dicobalt hexacarbonyl derivatives **6** and **7** via the Nicholas reaction (BF_3_·OEt_2_, CH_2_Cl_2_, 0 °C to rt).

entry	nucleoside	nucleophile	product	yield [%]

1	**4**	allyltrimethylsilane	**6a**	55
2	**4**	1,3,5-trimethoxybenzene	**6b**	89
3	**4**	acetophenone trimethylsilyl enol ether	**6c**	49
4	**5**	allyltrimethylsilane	**7a**	38
5	**5**	allyltrimethylsilane	**7a**	37 (46)^a,b^
6	**5**	1,3,5-trimethoxybenzene	**7b**	47
7	**5**	*N*-methylindole	**7d**	40

^a^Using SnCl_4_. ^b^Yield in parentheses is based on recovered starting material (brsm).

The reaction products were characterized by the disappearance of the formally diastereotopic propargylic methylene ^1^H NMR (CDCl_3_) spectral resonances (ca. 5.5 ppm in **4** and 4.8 ppm in **5**) and their reappearance upfield in the reaction products (i.e., 3.17 ppm in **6a** and 4.27 ppm in **6b**). In the ^13^C NMR spectra, the slightly broadened resonance of the metal carbonyl carbons at 199–200 ppm (199.4 ppm in both **6a** and **6b**) were characteristic of the product alkyne–Co_2_(CO)_6_ complexes. In the IR spectra, the series of intense metal–CO stretching bands between 2000–2100 cm^−1^ (e.g., **6a**, 2089, 2049, 2017 cm^−1^; **6b**, 2088, 2048, 2018 cm^−1^) dominated even the organic carbonyl bands.

## Conclusion

The Nicholas reaction, in which site of reactivity is well-defined and commonly free from formation of allenic byproducts, has been introduced into the repertoire of nucleosides modifications. The reaction of dicobalt hexacarbonyl propargylic alcohol uridine derivatives has been validated with diverse C-nucleophiles. By this means, alkene, arene, arylketo, and heterocyclic functions can be introduced onto metallo-nucleosides, preserving the dicobalt hexacarbonyl unit. This methodology allows for access in a divergent fashion to a variety of modified nucleosides with potential biological activity, and was shown to be viable for both 2'-deoxy- and regular uridines.

## Experimental

**General and instrumentation**. All NMR measurements were carried out on Bruker Avance III spectrometers operating for ^1^H NMR at 500 MHz, 600 MHz or 300 MHz and for ^13^C NMR at 125 MHz or 150 MHz, at 22 °C. Mass spectra were recorded on an Agilent 6520 Q-TOF LCMS (HRMS). FTIR spectra were recorded on ATI Mattson Infinity Series AR60, Thermo Scientific Nicolet 6700 ATR, or Bruker Alpha-P ATR spectrometers. All reactions were carried out under a nitrogen atmosphere and all products were stored in a freezer at −10 °C.

**3',5'-Di-*O*-acetyl-2'-deoxy-5-[3-(acetoxy)prop-1-yn-1-yl]uridine (2)**. A round-bottom flask was charged with 3',5'-di-*O*-acetyl**-**2'-deoxy-5-iodouridine (**1a**, 0.500 g, 1.14 mmol), Pd(PPh_3_)_4_ (0.066 g, 0.057 mmol), CuI (0.011 g, 0.057 mmol), DMF (10 mL), Et_3_N (396 µL, 2.85 mmol), and propargyl acetate (283 µL, 2.85 mmol). The reaction mixture was stirred at room temperature for 22 h. The solvent was removed by oil pump vacuum, and the residue was purified using silica gel column chromatography (230–400 mesh, eluent: 0→2% methanol in chloroform). The product was dried by oil pump vacuum for 2 h to give **2** as a white foam (0.405 g, 0.992 mmol, 87%). ^1^H NMR (500 MHz, DMSO-*d*_6_) δ 11.75 (s, 1H, N-H), 8.01 (s, 1H, H-6), 6.12 (t, *J* = 6.9 Hz, 1H, H-1'), 5.19–5.15 (m, 1H, H-3'), 4.87 (s, 2H, CH_2_), 4.27–4.23 (m, 2H, H-4', H-5'), 4.21–4.17 (m, 1H, H-5"), 2.52–2.47 (m, 1H, H-2'), 2.35–2.28 (m, 1H, H-2"), 2.07 (s, 3H, CH_3_), 2.05 (s, 6H, 2CH_3_); ^13^C NMR (125 MHz, DMSO-*d*_6_) δ 170.10, 170.03, 169.71, 161.30, 149.33, 144.40, 97.85, 87.05, 84.90, 81.48, 78.52, 73.66, 63.53, 52.20, 36.17, 20.76, 20.54, 20.46; IR (cm^–1^, KBr) 3442 m, 3389 m, 2987 m, 2823 m, 1701 s, 1627 s, 1467 m, 1288 m, 1052 m; TOF–ESI^+^–MS (*m*/*z*): [M + Na]^+^ calcd for C_18_H_20_N_2_NaO_9,_ 431.1061; found, 431.1064.

**2',3',5'-Tri-*O*-acetyl-5-(3-methoxyprop-1-yn-1-yl)uridine (3)**. A round-bottom flask was charged with 2',3',5'-tri-*O*-acetyl**-**5-iodouridine (**1b**, 0.500g, 1.01 mmol), Pd(PPh_3_)_4_ (0.058 g, 0.050 mmol), CuI (0.010 g, 0.050 mmol), DMF (10 mL), Et_3_N (351 µL, 2.52 mmol), and methyl propargyl ether (212 µL, 2.52 mmol). The reaction mixture was stirred at room temperature for 22 h. The solvent was removed by oil pump vacuum, and the residue was purified using silica gel column chromatography (230–400 mesh, eluent: 0→2% methanol in chloroform). The product was dried by oil pump vacuum for 2 h to give **3** as a white foam (0.270 g, 0.616 mmol, 61%). ^1^H NMR (500 MHz, CDCl_3_) δ 8.78 (s, 1H, N-H), 7.79 (s, 1H, H-6), 6.08–6.06 (m, 1H, H-1'), 5.35–5.30 (m, 2H, H-3', H-4'), 4.40–4.37 (m, 1H, H-2'), 4.37–4.35 (m, 2H, H-5', H-5"), 4.28 (s, 2H, CH_2_), 3.40 (s, 3H, OCH_3_), 2.21 (s, 3H, CH_3_), 2.12 (s, 3H, CH_3_), 2.11 (s, 3H, CH_3_); ^13^C NMR (125 MHz, CDCl_3_) δ 170.13, 169.65, 169.55, 160.68, 142.06, 100.66, 90.35, 87.46, 80.19, 75.44, 73.20, 70.01, 62.91, 60.30, 57.88, 51.08, 20.83, 20.54, 20.45; IR (cm^–1^, KBr) 3208 br w, 3082 br w, 2938 br w, 2823 br w, 1743 s, 1692 vs, 1628 m, 1453 m, 1214 vs, 1092 s; TOF–ESI^+^–MS (*m*/*z*): [M + Na]^+^ calcd for C_19_H_22_N_2_NaO_10_, 461.1167; found, 461.1171.

**General procedure for the synthesis of hexacarbonyl dicobalt 5-alkynyluridines (4 or 5):** A round-bottom flask was charged under a nitrogen atmosphere with Co_2_(CO)_8_ (0.222 g, 0.650 mmol), alkynyl nucleoside **2** or **3** (0.500 mmol), and THF (10 mL). The mixture was stirred at room temperature (22 °C) for 1 h. The solvent was removed by rotary evaporation. Silica gel column chromatography (230–400 mesh, eluent: chloroform) gave reddish-brown compounds **4** or **5**.

**Hexacarbonyl dicobalt 3',5'-di-*O*-acetyl-2'-deoxy-5-[3-(acetoxy)prop-1-yn-1-yl]uridine (4)**. From alkynyl nucleoside **2** (0.204 g, 0.500 mmol); brown foam (0.305 g, 0.440 mmol, 88%); ^1^H NMR (600 MHz, CDCl_3_) δ 9.32 (s, 1H, NH), 7.83 (s, 1H, H-6), 6.26–6.22 (m, 1H, H-1'), 5.57–5.47 (m, 2H, CH_2_), 5.23–5.20 (m, 1H, H-3'), 4.41–4.37 (m, 1H, H-4'), 4.32–4.29 (m, 1H, H-5'), 4.28–4.24 (m, 1H, H-5"), 2.66–2.61 (m, 1H, H-2'), 2.17–2.11 (m, 7H, H-2", 2CH_3_), 2.07 (s, 3H, CH_3_); ^13^C NMR (150 MHz, CDCl_3_) δ 198.71, 170.73, 170.29, 170.26, 160.23, 149.43, 138.26, 113.71, 94.82, 85.91, 82.58, 79.29, 74.03, 65.45, 63.65, 37.93, 20.90, 20.60, 20.54; IR (cm^–1^, KBr) 3356 br m, 3089 br w, 2960 br w, 2093 m, 2056 s, 2024 br s, 1736 vs, 1638 m, 1561 m, 1406 m, 1228 vs, 1024 s; TOF–ESI^+^–MS (*m*/*z*): [M + Na]^+^ calcd for C_24_H_20_Co_2_N_2_NaO_15_, 716.9420; found, 716.9426.

**Hexacarbonyl dicobalt 2',3',5'-tri-*O*-acetyl-5-(3-methoxyprop-1-yn-1-yl)uridine (5)**. From alkynyl nucleoside **3** (0.219 g, 0.500 mmol); brown foam (0.279 g, 0.385 mmol, 77%); ^1^H NMR (500 MHz, CDCl_3_) δ 9.56 (s, 1H, NH), 7.74 (s, 1H, H-6), 6.10–6.00 (m, 1H, H-1'), 5.41–5.30 (m, 2H, H-3', H-4'), 4.79 (m, 2H, CH_2_), 4.41–4.27 (m, 3H, H-5', H-5", H-2'), 3.54 (s, 3H, OCH_3_), 2.15 (s, 3H, CH_3_), 2.12 (s, 3H, CH_3_), 2.10 (s, 3H, CH_3_); ^13^C NMR (125 MHz, CDCl_3_) δ 198.97, 170.42, 169.66, 160.56, 150.01, 138.54, 114.36, 96.13, 87.92, 79.99, 79.19, 73.53, 72.44, 70.30, 63.34, 59.12, 20.69, 20.59, 20.44; IR (cm^–1^, KBr) 3234 br w, 2991 w, 2092 m, 2051 s, 2004 vs, 1746 m, 1688 m, 1447 m, 1214 br m, 1094 m, 750 vs; TOF–ESI^+^–MS (*m*/*z*): [M + Na]^+^ calcd for C_25_H_22_Co_2_N_2_NaO_16_, 746.9526; found, 746.9536.

**Hexacarbonyl dicobalt 3',5'-di-*O*-acetyl-2'-deoxy-5-(hex-5-en-1-yn-1-yl)uridine (6a)**. To a solution of nucleoside complex **4** (0.0206 g, 29.7 μmol) in CH_2_Cl_2_ (5 mL) at 0 °C was added allyltrimethylsilane (25 μL, 0.16 mmol) and BF_3_·OEt_2_ (15 μL, 0.12 mmol). The solution was stirred over 12 h with gradual warming to room temperature, at which time starting material consumption was complete, as evidenced by TLC (1:1 petroleum ether/EtOAc). Then, NH_4_Cl (saturated aq, 0.25 mL) and NaHCO_3_ (saturated aq, 0.25 mL) were added, followed by MgSO_4_. The mixture was filtered through a plug of silica gel and washed with EtOAc. Concentration of the crude reaction product and purification by flash chromatography (2:1→3:2 petroleum ether/EtOAc) afforded **6a** as a red-brown oil (0.0110 g, 16.3 μmol, 55%). ^1^H NMR (500 MHz, CDCl_3_) δ 8.85 (br s, 1H), 7.72 (s, 1H), 6.24 (dd, *J* = 8.0 Hz, 5.4 Hz, 1H), 5.93 (m, 1H), 5.22 (d, *J* = 6.3 Hz, 1H), 5.15 (d, *J* = 17.2 Hz, 1H), 5.05 (d, *J* = 10.1 Hz, 1H), 4.37 (dd, *J* = 11.6, 5.1 Hz, 1H), 4.30 (br s, 1H), 4.25 (dd, *J* = 11.6, 3.6 Hz, 1H), 3.17 (apparent t, *J* = 7.9 Hz, 2H), 2.61 (dd, *J* = 14.0, 4.6 Hz, 1H), 2.43 (dt, *J* = 8.1, 7.0 Hz, 2H), 2.13 (s, 3H), 2.12 (obscured, 1H), 2.08 (s, 3H); ^13^C NMR (125 MHz, CDCl_3_) δ 199.4, 170.3, 159.9, 149.4, 137.2, 115.6, 114.6, 103.6, 85.7, 82.4, 81.8, 74.0, 63.7, 37.9, 35.6, 33.8, 20.9, 20.6; IR (neat, ATR) 3197, 3077, 2967, 2089, 2049, 2017, 1747, 1714, 1691, 1587 cm^−1^; ESI^+^–MS (*m*/*z*): [M]^+^ calcd for C_25_H_22_Co_2_N_2_O_13_, 698.9684; found, 698.9689.

**Hexacarbonyl dicobalt 3',5'-di-*O*-acetyl-2'-deoxy-5-[3-(2,4,6-trimethoxyphenyl)prop-1-yn-1-yl]uridine (6b)**. To a solution of nucleoside complex **4** (0.0210 g, 30.3 μmol) in CH_2_Cl_2_ (5 mL) at 0 °C was added 1,3,5-trimethoxybenzene (0.0286 g, 0.170 mmol) and BF_3_·OEt_2_ (17 μL, 0.14 mmol). The solution was stirred for 0.5 h at 0 °C, followed by 1.5 h at room temperature. Then, NH_4_Cl (saturated aq, 0.25 mL) and NaHCO_3_ (saturated aq, 0.25 mL) were added, followed by a conventional extractive workup (CH_2_Cl_2_). Purification by preparative TLC (2:1 hexanes/EtOAc, 2 developments) afforded **6b** (0.0218 g, 26.9 μmol, 89%) as a red-brown oil. ^1^H NMR (500 MHz, CDCl_3_) δ 8.95 (br s, 1H), 7.74 (s, 1H), 6.27 (dd, *J* = 8.8, 5.4 Hz, 1H), 6.14 (s, 2H), 5.22 (d, *J* = 6.5 Hz, 1H), 4.36 (dd, *J* = 11.6, 4.9 Hz, 1H), 4.29 (m, 1H), 4.27 (s, 2H), 4.25 (dd, *J* = 11.6, 3.7 Hz, 1H), 3.82 (s, 3H), 3.77 (s, 6H), 2.60 (ddd, *J* = 14.2, 5.3, 1.3 Hz, 1H), 2.15 (m, 1H), 2.13 (s, 3H), 2.07 (s, 3H); ^13^C NMR (125 MHz, CDCl_3_) δ 199.4, 170.3, 160.3, 160.1, 158.9, 149.6, 136.2, 115.1, 108.7, 104.1, 90.0, 85.7, 82.3, 81.1, 74.1, 63.8, 55.3, 54.7, 37.7, 26.5, 20.9, 20.6; IR (neat, ATR) 3200, 2997, 2962, 2088, 2048, 2018, 1746, 1711, 1664, 1598 cm^−1^; ESI^+^–MS (*m*/*z*): [M]^+^ calcd for C_31_H_28_Co_2_N_2_O_16_, 825.0000; found, 825.0002.

**Hexacarbonyl dicobalt 3',5'-di-*O*-acetyl-2'-deoxy-5-(5-oxo-5-phenylhex-1-yn-1-yl)uridine (6c)**. To a solution of nucleoside complex **4** (0.0212 g, 30.6 μmol) in CH_2_Cl_2_ (5 mL) at 0 °C was added acetophenone trimethylsilyl enol ether (trimethyl(1-phenylvinyloxy)silane, 0.039 g, 0.20 mmol) and BF_3_·OEt_2_ (16 μL, 0.13 mmol). The solution was stirred over 12 h with gradual warming to room temperature. Then, NH_4_Cl (saturated saturated aq, 5 drops) and NaHCO_3_ (saturated aq, 5 drops) were added, followed by a conventional extractive workup (CH_2_Cl_2_). Purification by preparative TLC (3:2 hexanes/EtOAc, 2 developments) afforded **6c** as brown oil (0.0108 g, 15.0 μmol, 49%). ^1^H NMR (500 MHz, CDCl_3_) δ 8.68 (s, 1H), 8.00 (d, *J* = 7.9 Hz, 2H), 7.83 (s, 1H), 7.58 (t, *J* = 7.4 Hz, 1H), 7.48 (apparent t, *J* = 7.7 Hz, 2H), 6.26 (dd, *J* = 8.7, 5.4 Hz, 1H), 5.23 (d, *J* = 6.4 Hz, 1H), 4.39 (dd, *J* = 11.7, 5.4 Hz, 1H), 4.31 (m, 1H), 4.26 (dd, *J* = 11.7, 3.8 Hz, 1H), 3.52 (m, 2H), 3.42 (t, *J* = 7.1 Hz, 2H), 2.71 (dd, *J* = 13.7, 4.8 Hz, 1H), 2.19 (m, 1H), 2.13 (s, 3H), 2.08 (s, 3H); ^13^C NMR (125 MHz, CDCl_3_) δ 199.2, 198.3, 170.29, 170.26, 159.9, 149.3, 137.5, 136.6, 133.2, 128.7, 128.0, 114.1, 102.4, 85.6, 82.5, 81.8, 74.0, 63.7, 40.2, 37.8, 28.9, 20.9, 20.6; IR (neat, ATR) 3208, 2956, 2926, 2089, 2050, 2018, 1746, 1715, 1688, 1597 cm^−1^; ESI^+^–MS (*m*/*z*): [M + Na]^+^ calcd for C_30_H_25_Co_2_N_2_NaO_14_, 776.9789; found, 776.9788.

**Hexacarbonyl dicobalt 2',3',5'-tri-*O*-acetyl-5-(hex-5-en-1-yn-1-yl)uridine (7a)**. To a solution of nucleoside complex **5** (20.6 mg, 28.4 μmol) and allyltrimethylsilane (100 μL, 0.629 mmol) at 0 °C was added SnCl_4_ (90 μL, 1.0 M, 0.090 mmol). The solution was stirred for 1 h at 0 °C, followed by 2 h at rt. Then, NH_4_Cl (saturated aq, 5 drops) and NaHCO_3_ (saturated aq, 5 drops) were added, and a conventional extractive workup was performed (CH_2_Cl_2_). Preparative TLC (3:2 petroleum ether/EtOAc) afforded, in order of elution, **7a** (7.8 mg, 11 μmol, 37% yield, 46% brsm) and recovered **5** (3.7 mg, 5.1 μmol, 18% recovery). **7a**: ^1^H NMR (300 MHz, CDCl_3_) δ 8.60 (s, 1H), 7.55 (s, 1H), 6.00 (d, *J* = 5.4 Hz, 1H), 5.94 (m, 1H), 5.30–5.40 (m, 2H), 5.14 (d, *J* = 17.1 Hz, 1H), 5.06 (d, *J* = 10.2 Hz, 1H), 4.23–4.43 (m, 3H), 3.17 (apparent t, *J* = 7.9 Hz, 2H), 2.43 (m, 2H), 2.16 (s, 3H), 2.13 (s, 3H), 2.10 (s, 3H); ^13^C NMR (125 MHz, CDCl_3_) δ 199.2, 170.3, 169.5, 159.6, 149.4, 137.9, 137.2, 115.7, 115.2, 103.6, 88.0, 80.3, 80.1, 72.4, 70.3, 63.3, 35.6, 33.8, 20.6, 20.5, 20.4; IR (neat, ATR) 3219, 2956, 2924, 2089, 2049, 2014, 1749, 1718, 1692 cm^−1^; ESI^+^–MS (*m*/*z*): [M + Na]^+^ calcd for C_27_H_24_Co_2_N_2_NaO_15_, 756.9738; found, 756.9742.

**Hexacarbonyl dicobalt 2',3',5'-tri-*O*-acetyl-5-[3-(2,4,6-trimethoxyphenyl)prop-1-yn-1-yl)]uridine (7b)**. To a solution of nucleoside complex **5** (20.4 mg, 28.2 μmol) and 1,3,5-trimethoxybenzene (23.6 mg, 140 μmol) in CH_2_Cl_2_ (5 mL) at 0 °C was added BF_3_·OEt_2_ (11 μL, 87 μmol). The solution was stirred for 45 min at 0 °C, followed by 1 h at rt. Then, NH_4_Cl (saturated aq, 5 drops) and NaHCO_3_ (saturated aq, 5 drops) were added, and a conventional extractive workup was performed (CH_2_Cl_2_). Preparative TLC (3:2 hexanes/EtOAc) gave **7b** as viscous brown oil (11.3 mg, 13.2 μmol, 47%). ^1^H NMR (300 MHz, CDCl_3_) δ 8.64 (s, 1H), 7.56 (s, 1H), 6.14 (s, 2H), 5.92 (d, *J* = 5.7 Hz, 1H), 5.32–5.42 (m, 2H), 4.20–4.45 (m, 5H), 3.82 (s, 3H), 3.78 (s, 6H), 2.16 (s, 3H), 2.12 (s, 3H), 2.11 (s, 3H); ^13^C NMR (125 MHz, CDCl_3_) δ 199.3, 170.3, 169.5, 160.4, 159.9, 158.9, 149.6, 136.7, 115.6, 108.7, 103.9, 90.0, 87.9, 80.5, 80.1, 72.4, 70.3, 63.3, 55.3, 54.8, 25.6, 20.6, 20.5, 20.3; IR (neat, ATR) 3211, 2956, 2924, 2087, 2047, 2010, 1748, 1716, 1693, 1597 cm^−1^; ESI^+^–MS (*m*/*z*): [M + Na]^+^ calcd for C_33_H_30_Co_2_N_2_NaO_18_, 883.0055; found, 883.0077.

**Hexacarbonyl dicobalt 2',3',5'-tri-*O*-acetyl-5-[3-(1'-methylindol-3'-yl)prop-1-yn-1-yl)]uridine (7d)**. To a solution of nucleoside complex **5** (20.4 mg, 28.1 μmol) and *N*-methylindole (18.4 mg, 14.0 μmol) in CH_2_Cl_2_ (5 mL) at 0 °C was added BF_3_·OEt_2_ (14 μL, 0.11 mmol). The solution was stirred for 45 min at 0 °C, followed by rt for 45 min. Then, NH_4_Cl (saturated aq, 5 drops) and NaHCO_3_ (saturated aq, 5 drops) were added, and a conventional extractive workup was performed (CH_2_Cl_2_). Preparative TLC (3:2 hexanes/EtOAc) afforded **7d** as brown oil (9.2 mg, 11 μmol, 40%). ^1^H NMR (300 MHz, CDCl_3_) δ 8.39 (br s, 1H), 7.63 (d, *J* = 7.8 Hz, 1H), 7.46 (s, 1H), 7.29 (d, obscured, 1H), 7.24 (apparent t, *J* = 7.0 Hz, 1H), 7.12 (apparent dt, *J* = 1.0, 7.4 Hz, 1H), 6.96 (s, 1H), 5.84 (d, *J* = 5.4 Hz, 1H), 5.28–5.38 (m, 2H), 4.52 (s, 2H), 4.23–4.40 (m, 3H), 3.79 (s, 3H), 2.15 (s, 3H), 2.11 (s, 3H), 2.09 (s, 3H); ^13^C NMR (125 MHz, CDCl_3_) δ 199.1, 170.3, 169.53, 169.52, 158.9, 149.3, 138.1, 136.6, 127.7, 127.6, 121.6, 119.0, 118.7, 115.3, 113.8, 109.2, 106.6, 88.5, 81.3, 80.0, 72.4, 70.2, 63.2, 32.7, 29.6, 20.6, 20.5, 20.4; IR (neat, ATR) 3204, 2954, 2924, 2089, 2050, 2019, 1750, 1720, 1692 cm^−1^; ESI^+^–MS (*m*/*z*): [M + H]^+^ calcd for C_33_H_27_Co_2_N_3_O_15_, 823.0184; found, 823.0184.

## Supporting Information

File 1 ^1^H and ^13^C NMR spectra for compounds **2**, **3**, **4**, **5**, **6a**–**c**, and **7a**,**b**,**d**.
