# Supplementary material for: Extension of the 5-alkynyluridine side chain via C–C-bond formation in modified organometallic nucleosides using the Nicholas reaction
Source: Beilstein J Org Chem. 2020 Jan 2;16:1–8. doi: 10.3762/bjoc.16.1 (PMC6964655; doi:10.3762/bjoc.16.1)

BEILSTEIN JOURNAL OF ORGANIC CHEMISTRY

Supporting Information

for

Extension of the 5-alkynyluridine side chain via C–C-bond formation in modified organometallic nucleosides using the Nicholas reaction

Renata Kaczmarek, Dariusz Korczyński, James R. Green and Roman Dembinski

Beilstein J. Org. Chem. **2020,** *16,* 1–8. doi:10.3762/bjoc.16.1

^{1}H and ^{13}C NMR spectra for compounds 2, 3, 4, 5, 6a–c, and 7a,b,d

License and Terms: This is a supporting information file under the terms of the Creative Commons Attribution License (https://creativecommons.org/licenses/by/4.0). Please note that the reuse, redistribution and reproduction in particular requires that the authors and source are credited.
The license is subject to the *Beilstein Journal of Organic Chemistry* terms and conditions: (https://www.beilstein-journals.org/bjoc)

Table of Contents

^{1}H and ^{13}C NMR spectrum of **2**	S2
^{1}H and ^{13}C NMR spectrum of **3**	S4
^{1}H and ^{13}C NMR spectrum of **4**	S6
^{1}H and ^{13}C NMR spectrum of **5**	S8
^{1}H and ^{13}C NMR spectrum of **6a**	S10
^{1}H and ^{13}C NMR spectrum of **6b**	S12
^{1}H and ^{13}C NMR spectrum of **6c**	S14
^{1}H and ^{13}C NMR spectrum of **7a**	S16
^{1}H and ^{13}C NMR spectrum of **7b**	S18
^{1}H and ^{13}C NMR spectrum of **7d**	S20

^{1}H NMR spectrum for **2** (DMSO-d_6)

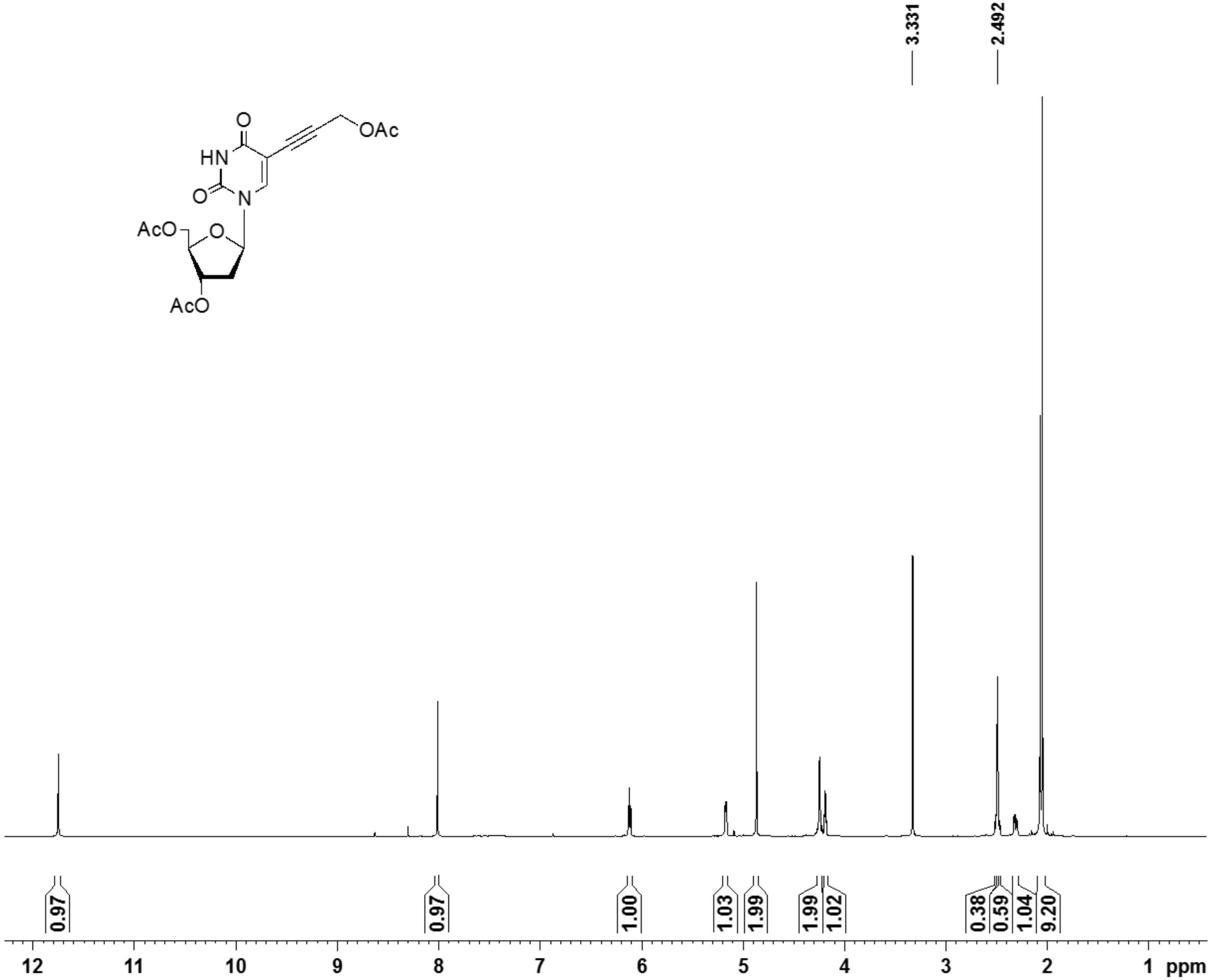

CBMiM PAN LODZ

**** NMR LAB ****

AV III 500 MHz

Tel:042 6803 307

Current Data Parameters
DATPATH C:\Bruker\TopSpin3.1\109
NAME kac12310_2017_600
EXPNO 1
PROCNO 1

F2 - Acquisition Parameters
Date_ 20171023
Time 11.31
INSTRUM AV_III_600
PROBHD 5 mm PABBO BB/
PULPROG zg30
TD 65536
SOLVENT DMSO
NS 64
DS 0
SWH 12335.526 Hz
FIDRES 0.188225 Hz
AQ 2.6563926 sec
RG 57
DW 40.533 usec
DE 6.50 usec
TE 294.9 K
D1 1.00000000 sec
TD0 1

======== CHANNEL f1 ========
SFO1 600.1339008 MHz
NUC1 1H
P1 11.00 usec
PLW1 25.00000000 W

F2 - Processing parameters
SI 65536
SF 600.1300135 MHz
WDW EM
SSB 0
LB 0.30 Hz
GB 0
PC 1.40

^{13}C NMR spectrum for **2** (DMSO-d_6)

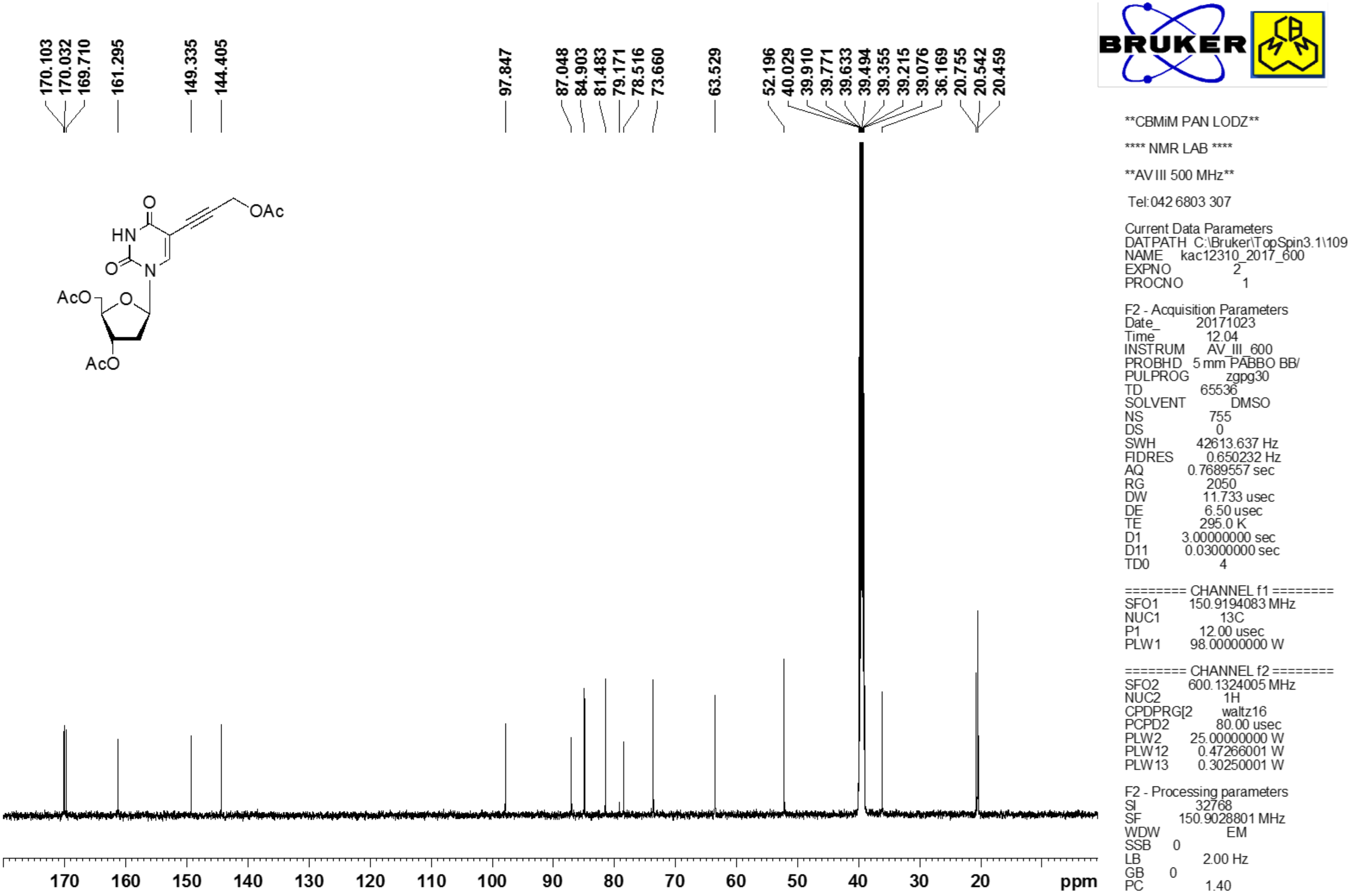

^{1}H NMR spectrum for **3** ($CDCl_3$)

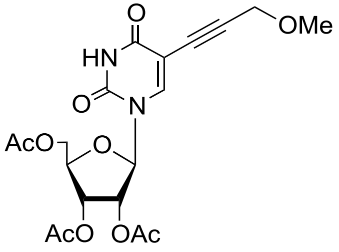

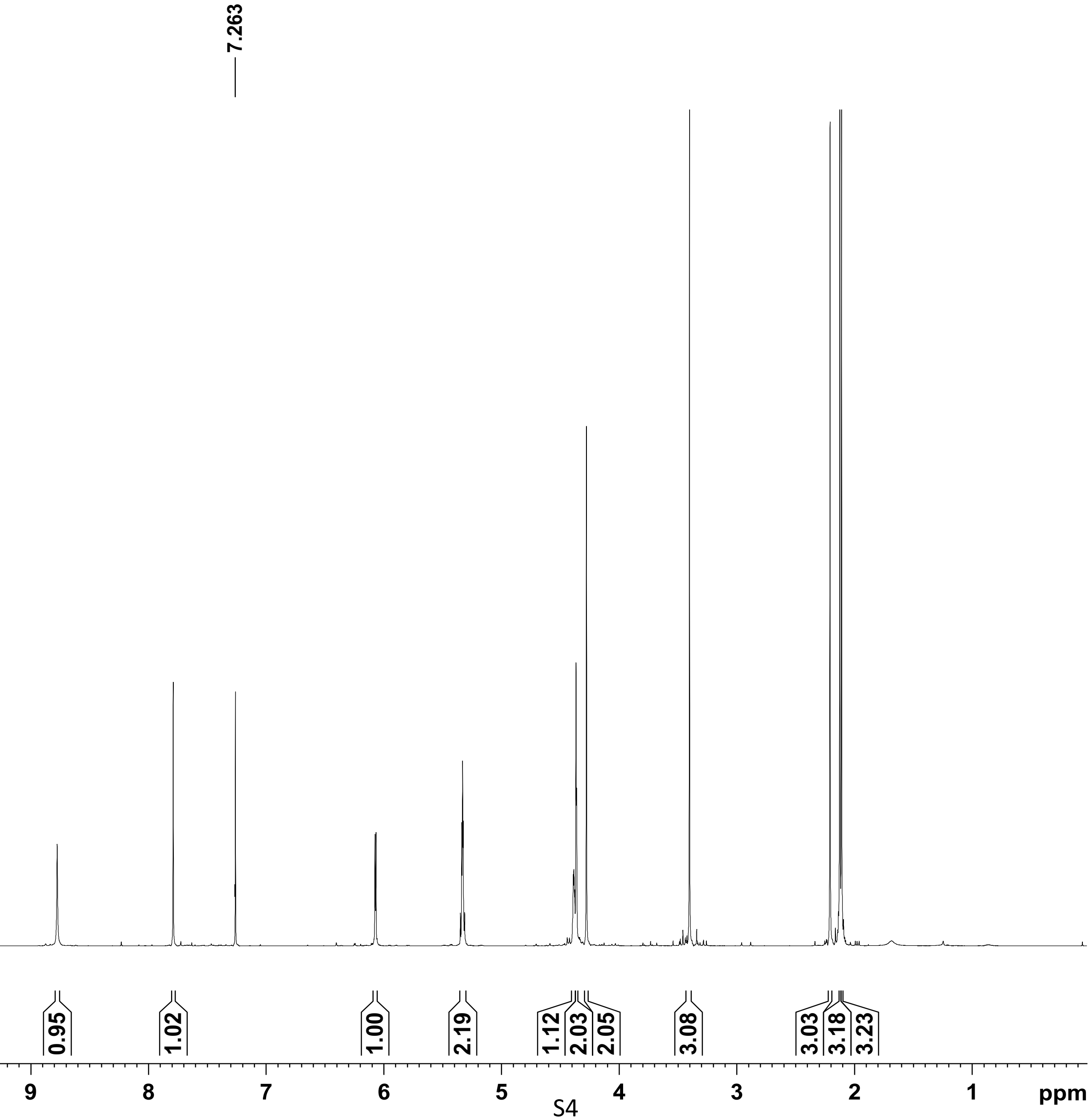

CBMiM PAN LODZ

**** NMR LAB ****

AV III 500 MHz

Tel:042 6803 307

Current Data Parameters
DATPATH C:\Bruker\TopSpin3.1\109
NAME kac10907_2019_500
EXPNO 1
PROCNO 1

F2 - Acquisition Parameters
Date_ 20190709
Time 10.29
INSTRUM AV_III_500
PROBHD 5 mm TXI 31P Z
PULPROG zg30
TD 65536
SOLVENT CDCl3
NS 24
DS 0
SWH 12335.526 Hz
FIDRES 0.188225 Hz
AQ 2.6563926 sec
RG 228
DW 40.533 usec
DE 6.50 usec
TE 295.0 K
D1 1.00000000 sec
TD0 1

======== CHANNEL f1 ========
SFO1 500.1330008 MHz
NUC1 1H
P1 9.66 usec
PLW1 9.19999981 W

F2 - Processing parameters
SI 65536
SF 500.1300236 MHz
WDW EM
SSB 0
LB 0.30 Hz
GB 0
PC 1.00

^{13}C NMR spectrum for **3** ($CDCl_3$)

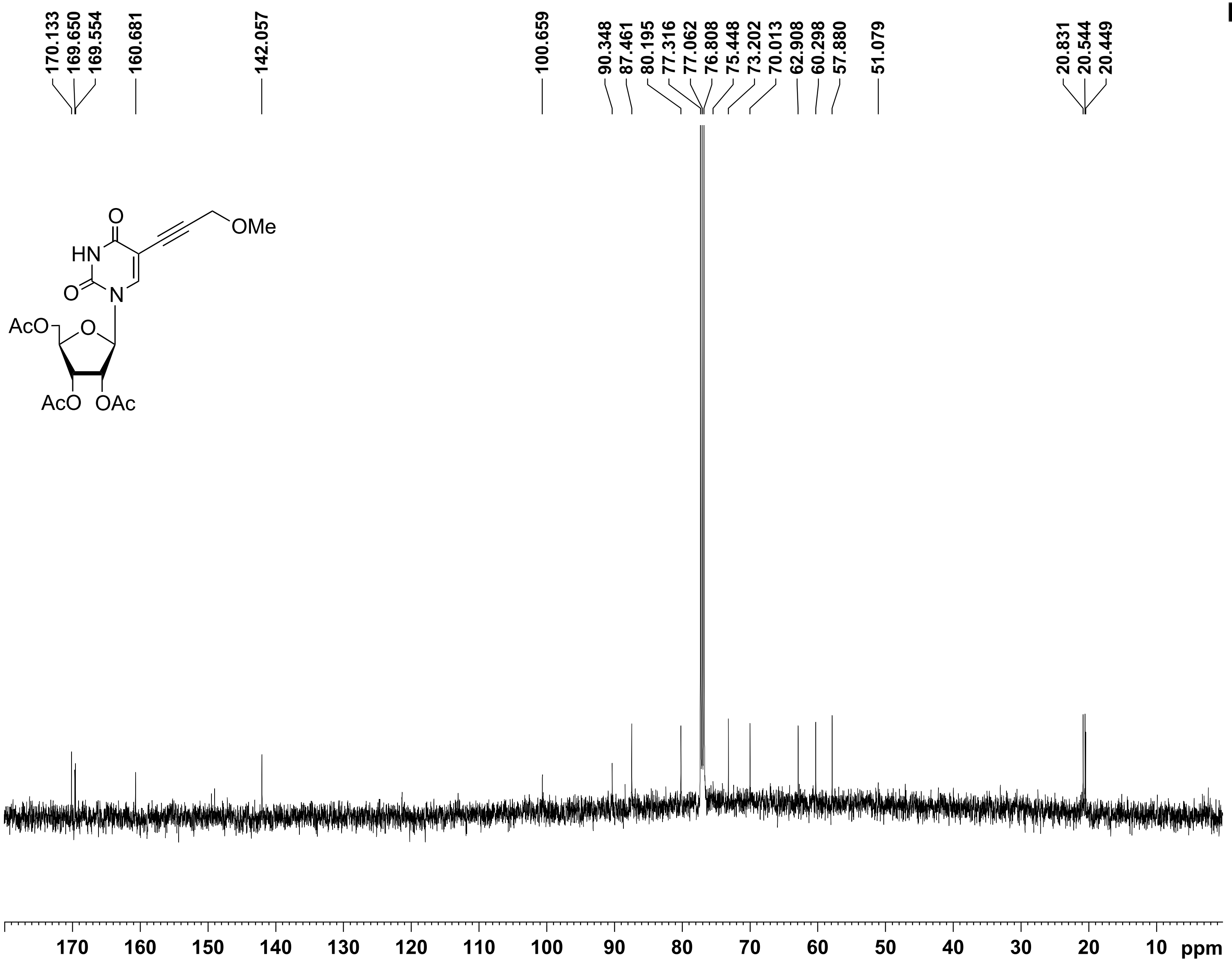

CBMiM PAN LODZ

**** NMR LAB ****

AV III 500 MHz

Tel:042 6803 307

Current Data Parameters
DATPATH C:\Bruker\TopSpin3.1\109
NAME kac10907_2019_500
EXPNO 2
PROCNO 1

F2 - Acquisition Parameters
Date_ 20190709
Time 10.55
INSTRUM AV_III_500
PROBHD 5 mm TXI 31P Z
PULPROG zgpg30
TD 65536
SOLVENT CDCl3
NS 512
DS 0
SWH 36057.691 Hz
FIDRES 0.550197 Hz
AQ 0.9087659 sec
RG 2050
DW 13.867 usec
DE 6.50 usec
TE 295.0 K
D1 2.00000000 sec
D11 0.03000000 sec
TD0 1

======== CHANNEL f1 ========
SFO1 125.7728788 MHz
NUC1 13C
P1 11.50 usec
PLW1 226.00000000 W

======== CHANNEL f2 ========
SFO2 500.1324005 MHz
NUC2 1H
CPDPRG[2 waltz16
PCPD2 100.00 usec
PLW2 9.00000000 W
PLW12 0.08398400 W
PLW13 0.08398400 W

F2 - Processing parameters
SI 32768
SF 125.7577890 MHz
WDW EM
SSB 0
LB 2.00 Hz
GB 0
PC 1.40

^{1}H NMR spectrum for **4** ($CDCl_3$)

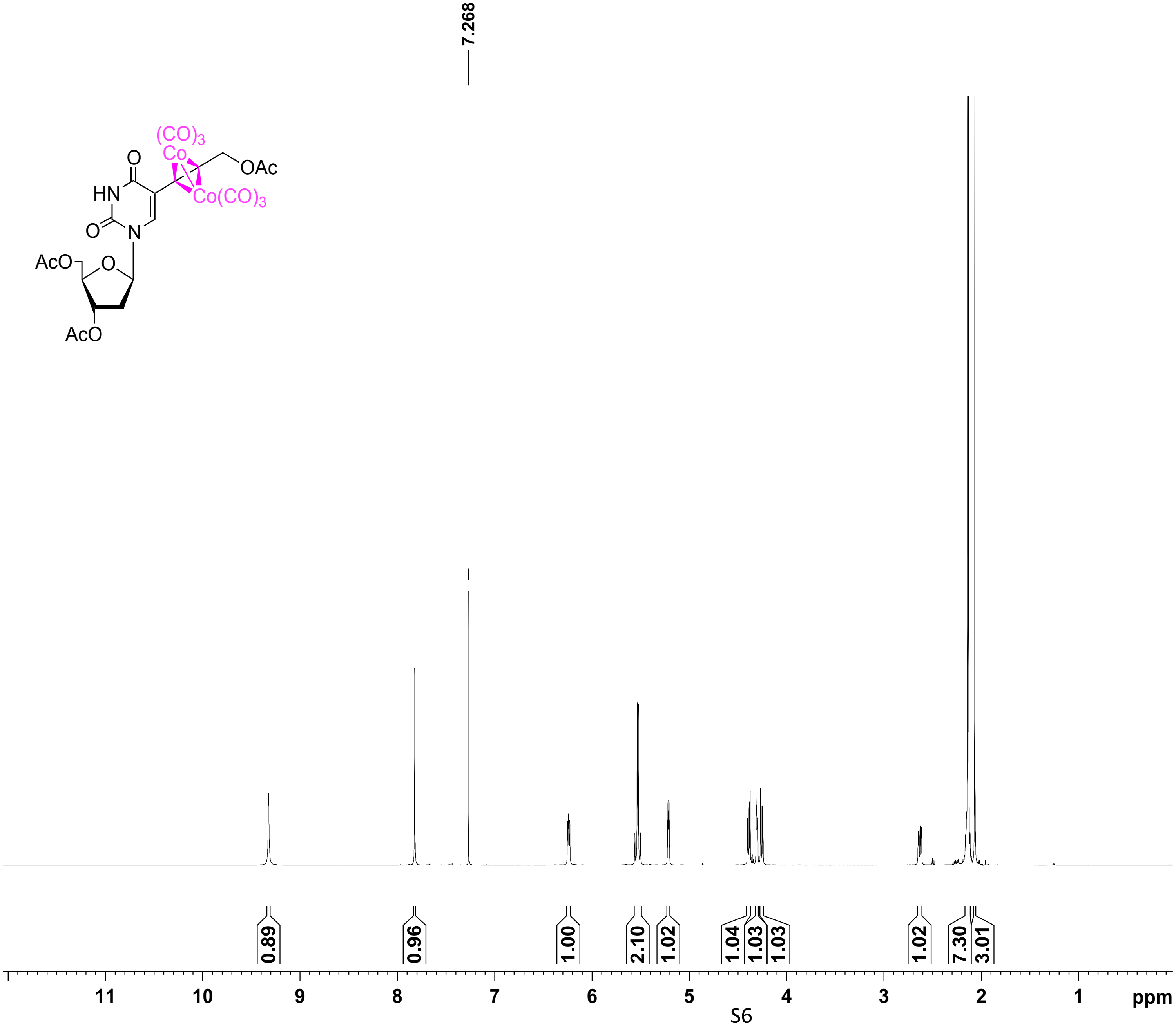

CBMiM PAN LODZ

**** NMR LAB ****

AV III 500 MHz

Tel:042 6803 307

Current Data Parameters
DATPATH C:\Bruker\TopSpin3.1\109
NAME kac12510_2017_600
EXPNO 1
PROCNO 1

F2 - Acquisition Parameters
Date_ 20171025
Time 11.32
INSTRUM AV_III_600
PROBHD 5 mm PABBO BB/
PULPROG zg30
TD 65536
SOLVENT CDCl3
NS 64
DS 0
SWH 12335.526 Hz
FIDRES 0.188225 Hz
AQ 2.6563926 sec
RG 57
DW 40.533 usec
DE 6.50 usec
TE 295.0 K
D1 1.00000000 sec
TD0 1

======== CHANNEL f1 ========
SFO1 600.1339008 MHz
NUC1 1H
P1 11.00 usec
PLW1 25.00000000 W

F2 - Processing parameters
SI 65536
SF 600.1300135 MHz
WDW EM
SSB 0
LB 0.30 Hz
GB 0
PC 1.40

^{13}C NMR spectrum for **4** ($CDCl_3$)

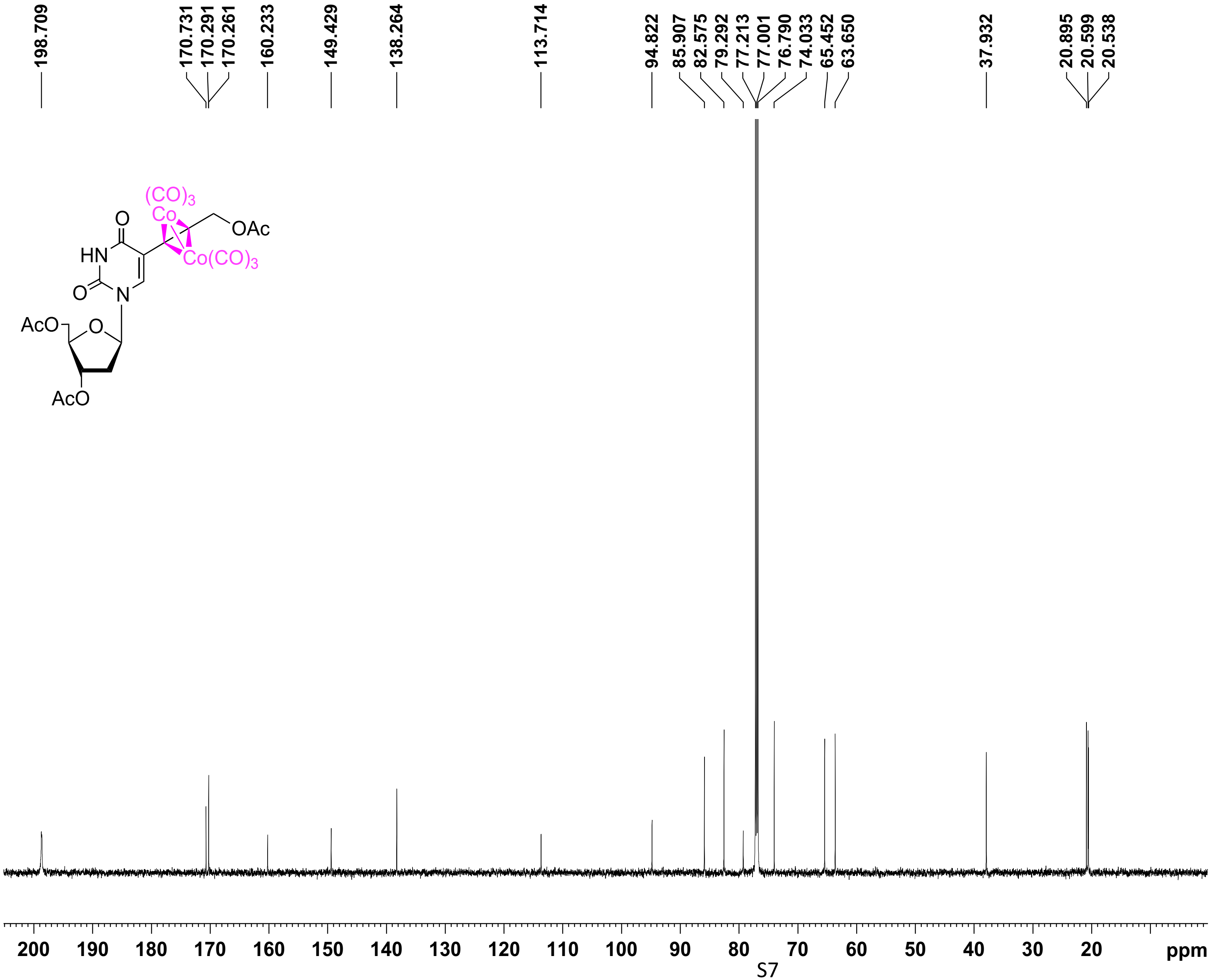

CBMiM PAN LODZ

**** NMR LAB ****

AV III 500 MHz

Tel:042 6803 307

Current Data Parameters
DATPATH C:\Bruker\TopSpin3.1\109
NAME kac12510_2017_600
EXPNO 2
PROCNO 1

F2 - Acquisition Parameters
Date_ 20171025
Time 11.58
INSTRUM AV_III_600
PROBHD 5 mm PABBO BB/
PULPROG zgpg30
TD 65536
SOLVENT CDCl3
NS 633
DS 0
SWH 42613.637 Hz
FIDRES 0.650232 Hz
AQ 0.7689557 sec
RG 2050
DW 11.733 usec
DE 6.50 usec
TE 295.1 K
D1 3.00000000 sec
D11 0.03000000 sec
TD0 2

======== CHANNEL f1 ========
SFO1 150.9194083 MHz
NUC1 13C
P1 12.00 usec
PLW1 98.00000000 W

======== CHANNEL f2 ========
SFO2 600.1324005 MHz
NUC2 1H
CPDPRG[2 waltz16
PCPD2 80.00 usec
PLW2 25.00000000 W
PLW12 0.47266001 W
PLW13 0.30250001 W

F2 - Processing parameters
SI 32768
SF 150.9028155 MHz
WDW EM
SSB 0
LB 2.00 Hz
GB 0
PC 1.40

^{1}H NMR spectrum for **5** ($CDCl_3$)

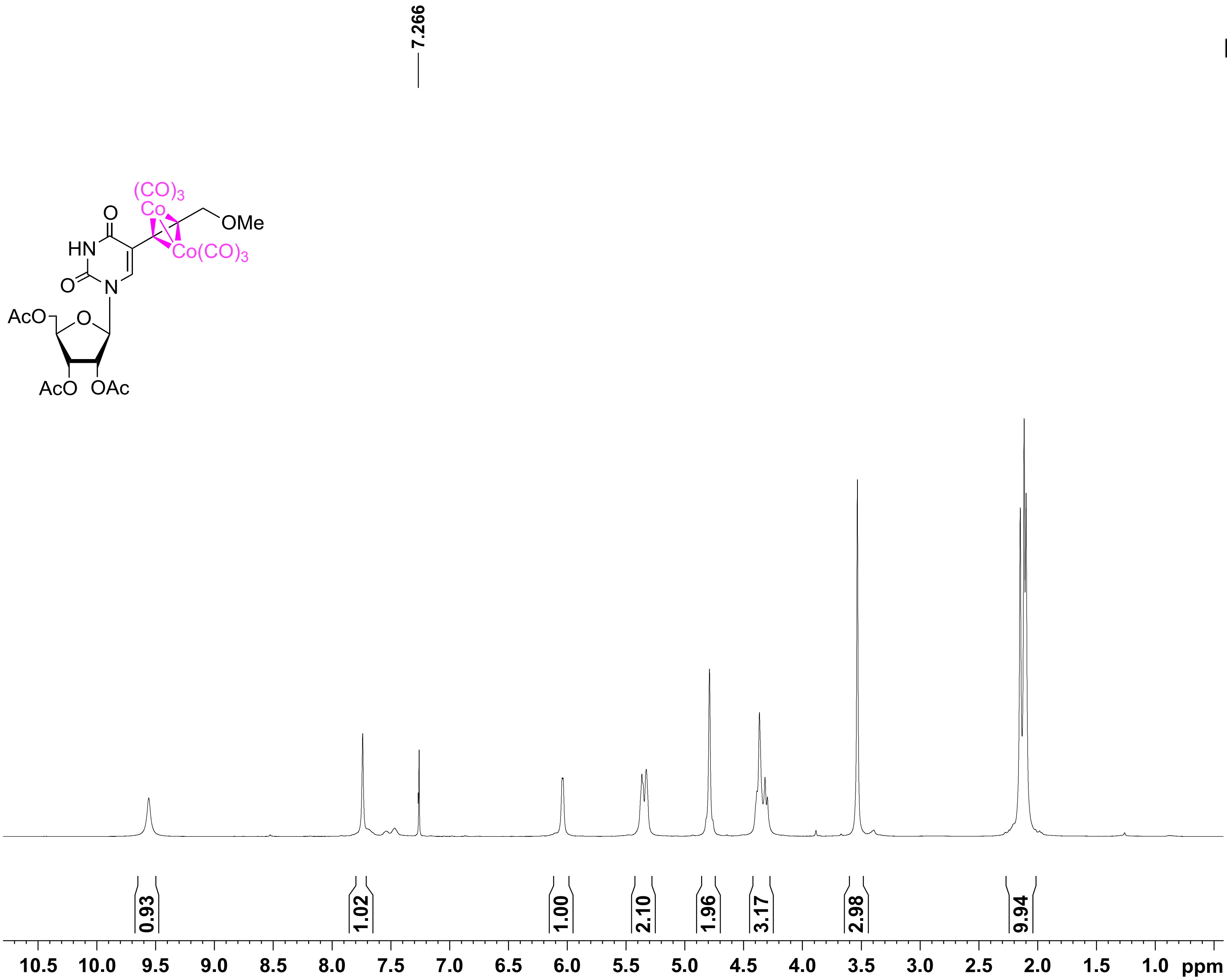

CBMiM PAN LODZ

**** NMR LAB ****

AV III 500 MHz

Tel:042 6803 307

Current Data Parameters
DATPATH C:\Bruker\TopSpin3.1\109
NAME kac10907_2019_500
EXPNO 3
PROCNO 1

F2 - Acquisition Parameters
Date_ 20190709
Time 10.58
INSTRUM AV_III_500
PROBHD 5 mm TXI 31P Z
PULPROG zg30
TD 65536
SOLVENT CDCl3
NS 24
DS 0
SWH 12335.526 Hz
FIDRES 0.188225 Hz
AQ 2.6563926 sec
RG 114
DW 40.533 usec
DE 6.50 usec
TE 295.0 K
D1 1.00000000 sec
TD0 1

======== CHANNEL f1 ========
SFO1 500.1330008 MHz
NUC1 1H
P1 9.66 usec
PLW1 9.19999981 W

F2 - Processing parameters
SI 65536
SF 500.1300236 MHz
WDW EM
SSB 0
LB 0.30 Hz
GB 0
PC 1.00

^{13}C NMR spectrum for **5** ($CDCl_3$)

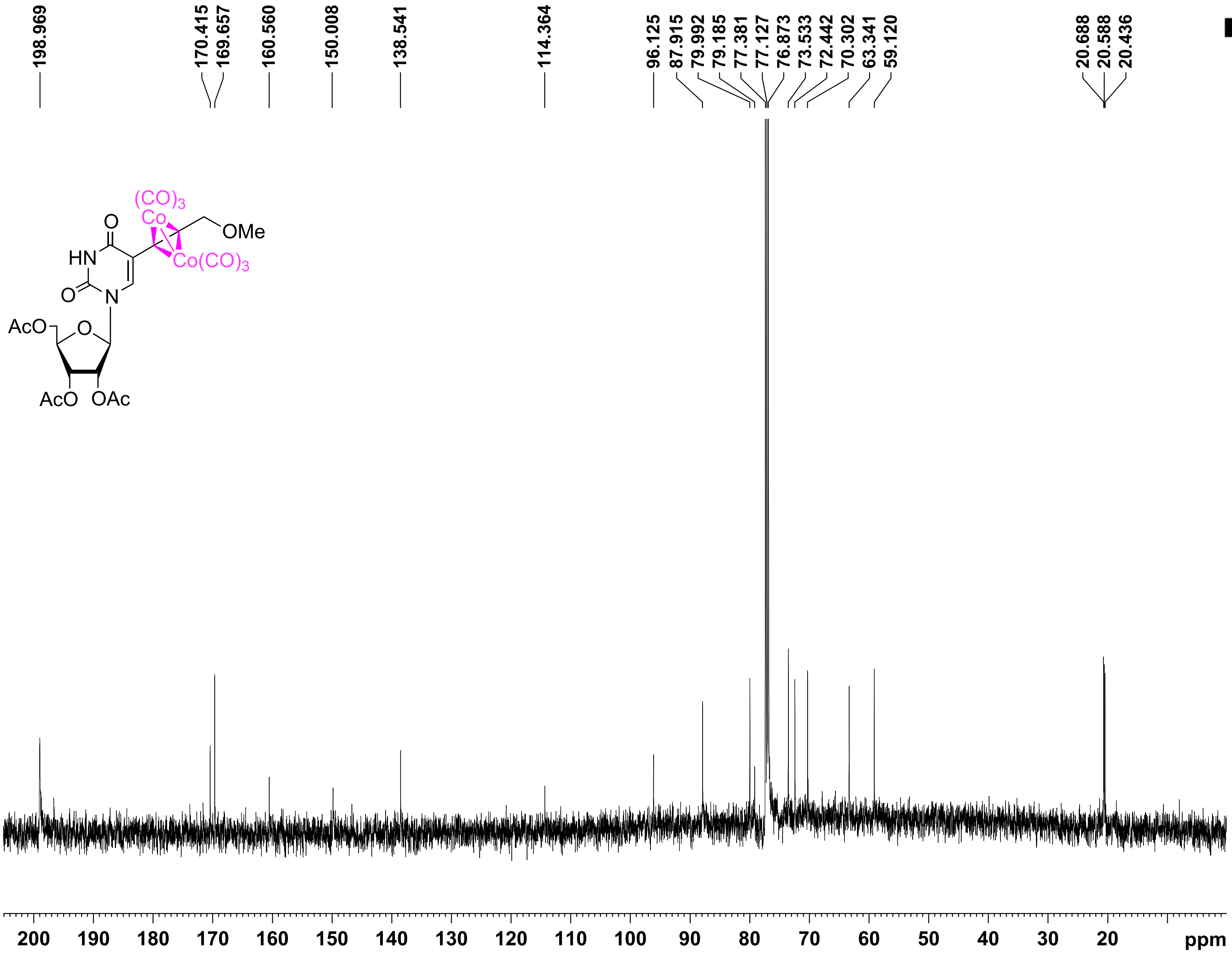

CBMiM PAN LODZ

**** NMR LAB ****

AV III 500 MHz

Tel:042 6803 307

Current Data Parameters
DATPATH C:\Bruker\TopSpin3.1\109
NAME kac10907_2019_500
EXPNO 4
PROCNO 1

F2 - Acquisition Parameters
Date_ 20190709
Time 11.07
INSTRUM AV_III_500
PROBHD 5 mm TXI 31P Z
PULPROG zgpg30
TD 65536
SOLVENT CDCl3
NS 512
DS 0
SWH 36057.691 Hz
FIDRES 0.550197 Hz
AQ 0.9087659 sec
RG 2050
DW 13.867 usec
DE 6.50 usec
TE 295.0 K
D1 2.00000000 sec
D11 0.03000000 sec
TD0 1

======== CHANNEL f1 ========
SFO1 125.7728788 MHz
NUC1 13C
P1 11.50 usec
PLW1 226.00000000 W

======== CHANNEL f2 ========
SFO2 500.1324005 MHz
NUC2 1H
CPDPRG[2 waltz16
PCPD2 100.00 usec
PLW2 9.00000000 W
PLW12 0.08398400 W
PLW13 0.08398400 W

F2 - Processing parameters
SI 32768
SF 125.7577890 MHz
WDW EM
SSB 0
LB 2.00 Hz
GB 0
PC 1.40

^{1}H NMR spectrum for **6a** ($CDCl_3$)

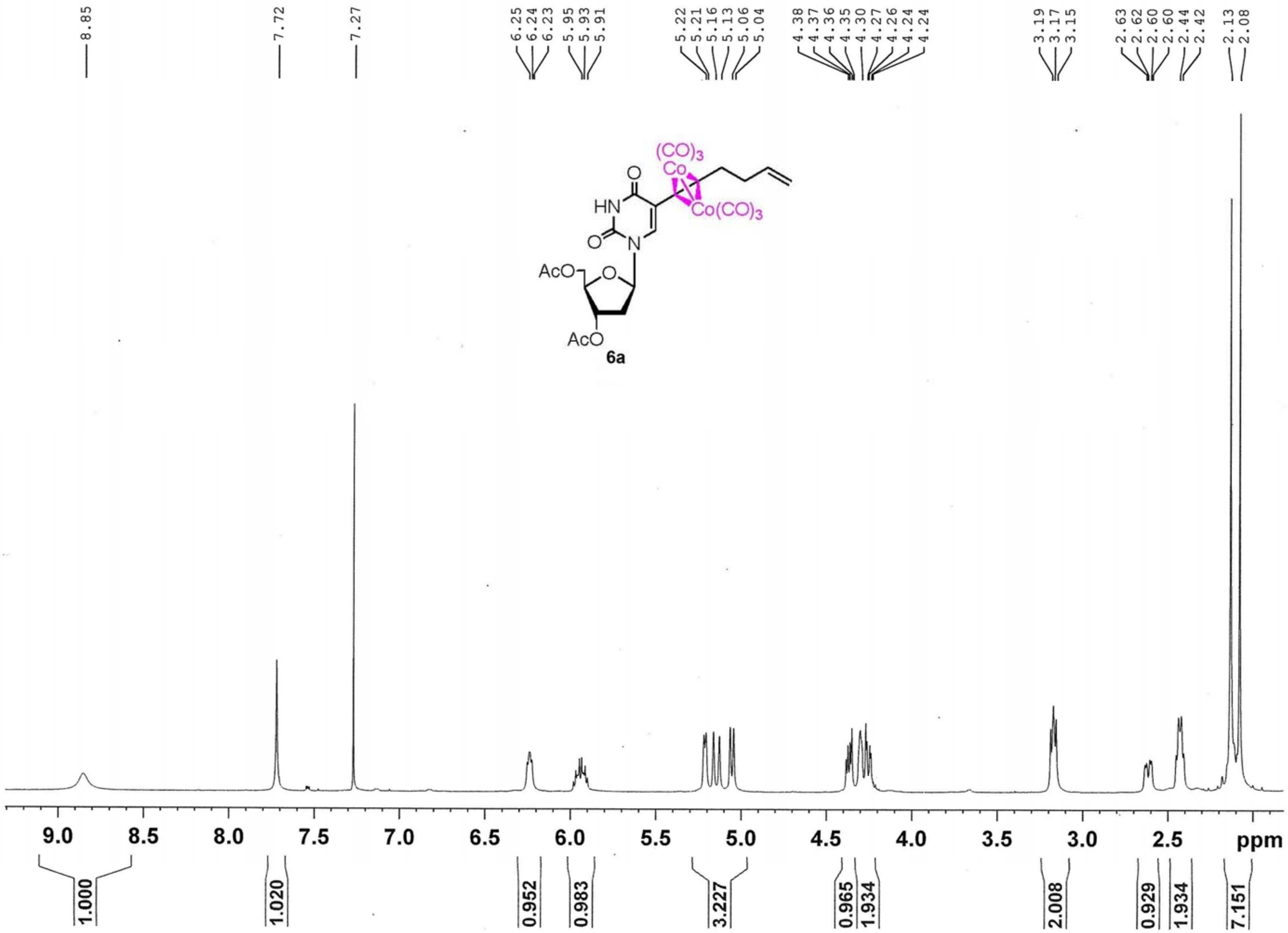

^{13}C NMR spectrum for **6a** ($CDCl_3$)

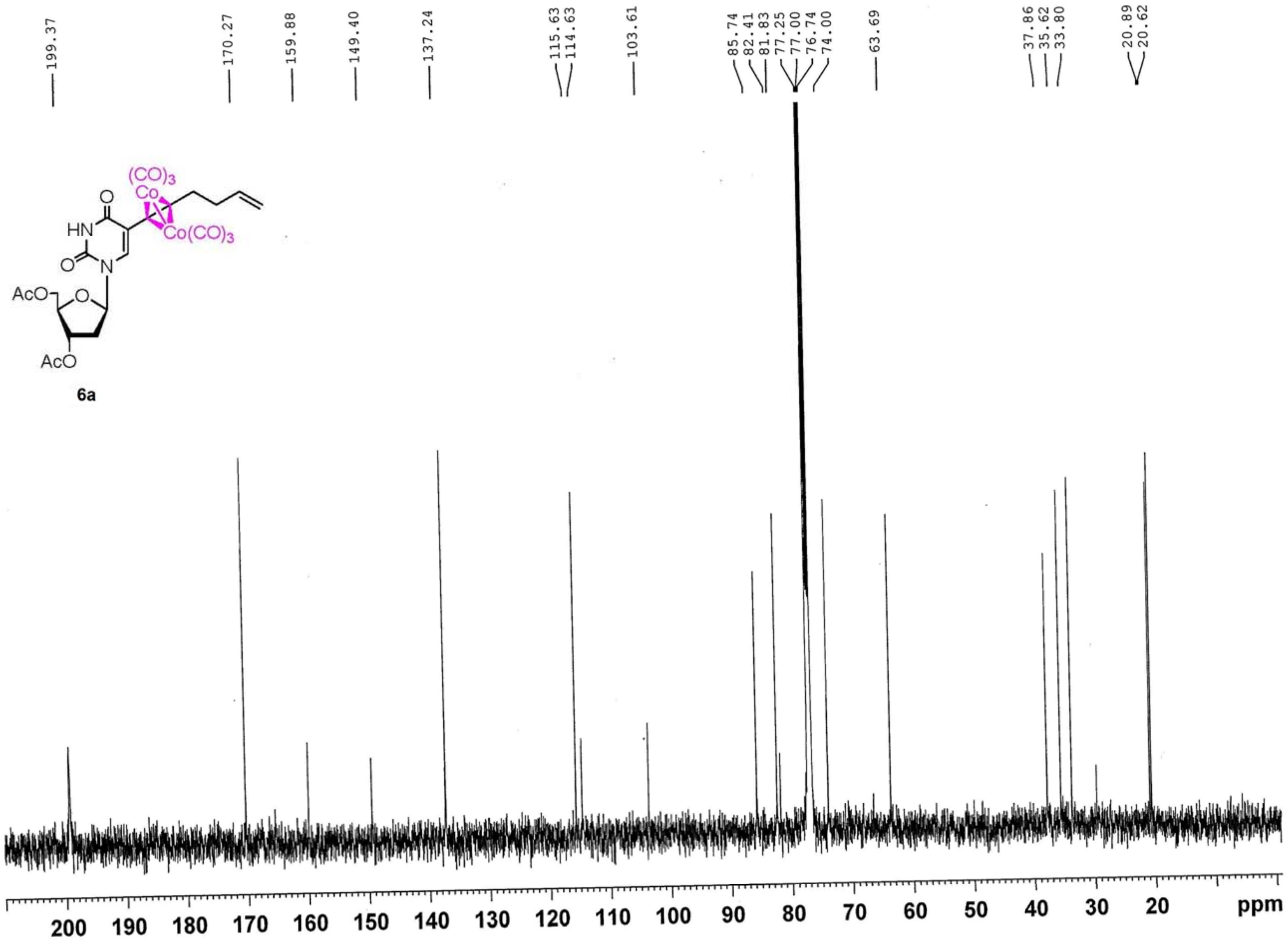

^{1}H NMR spectrum for **6b** ($CDCl_3$)

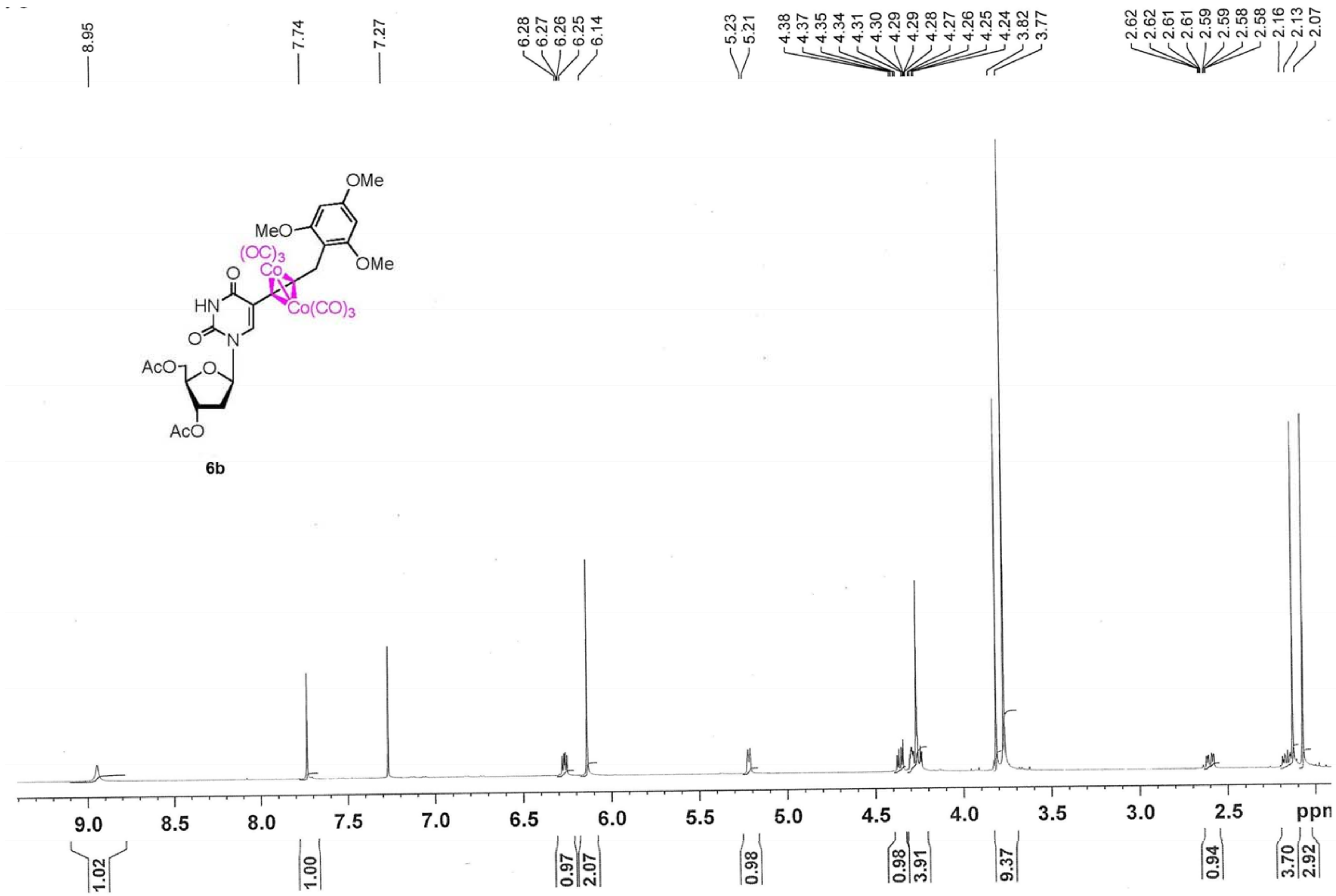

^{13}C NMR spectrum for **6b** ($CDCl_3$)

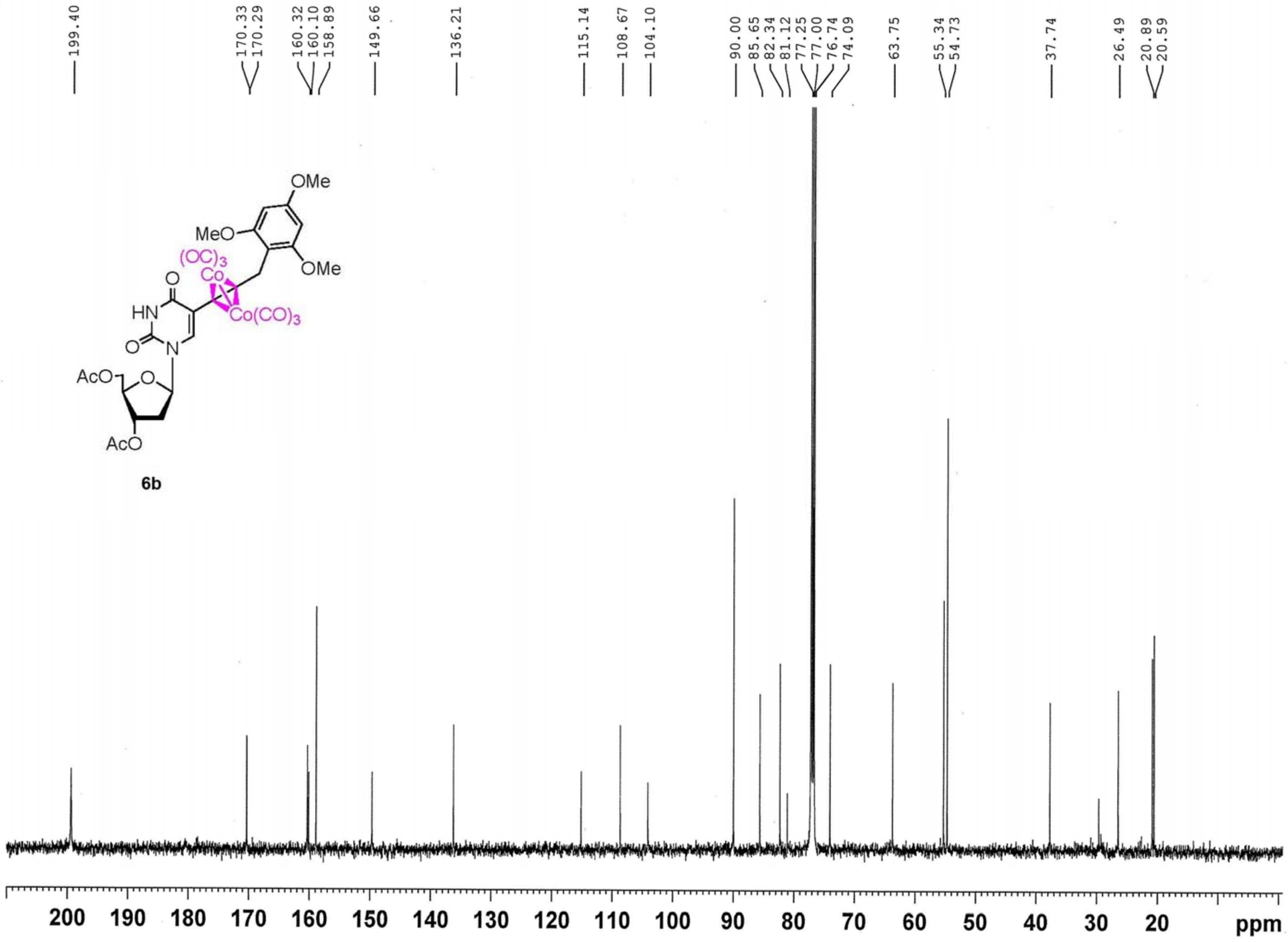

^{1}H NMR spectrum for **6c** ($CDCl_3$)

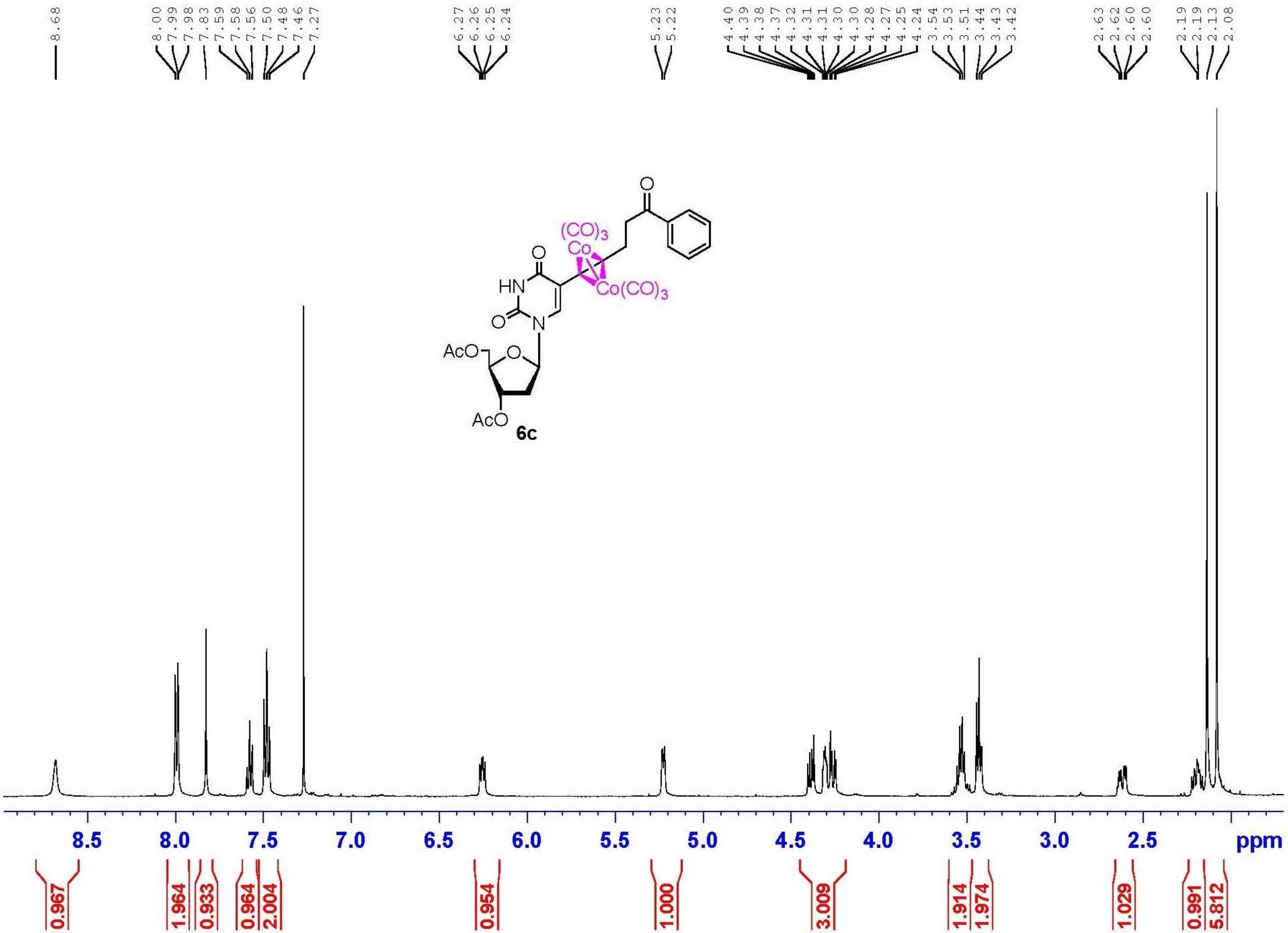

^{13}C NMR spectrum for **6c** ($CDCl_3$)

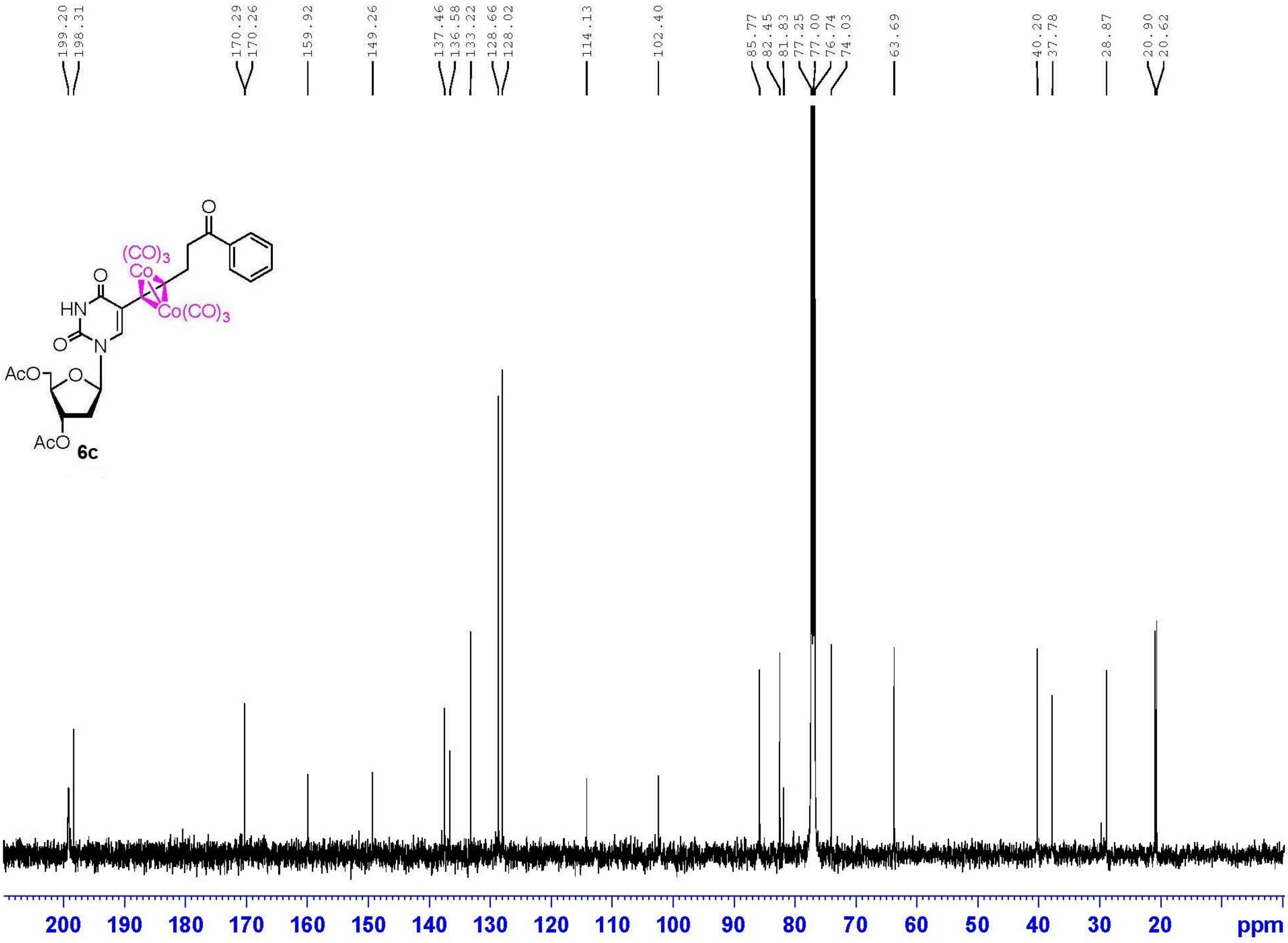

^{1}H NMR spectrum for **7a** ($CDCl_3$)

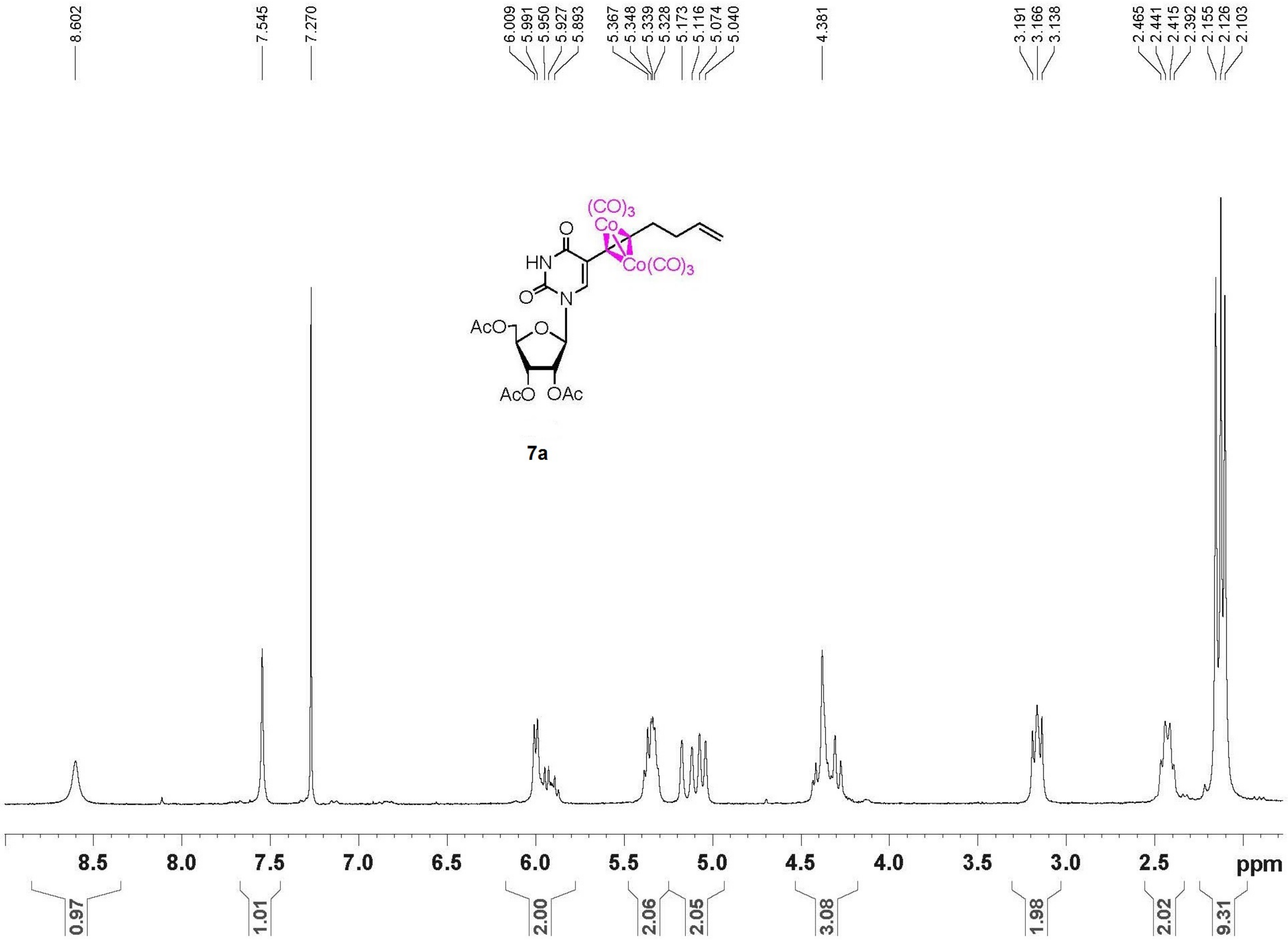

^{13}C NMR spectrum for **7a** ($CDCl_3$)

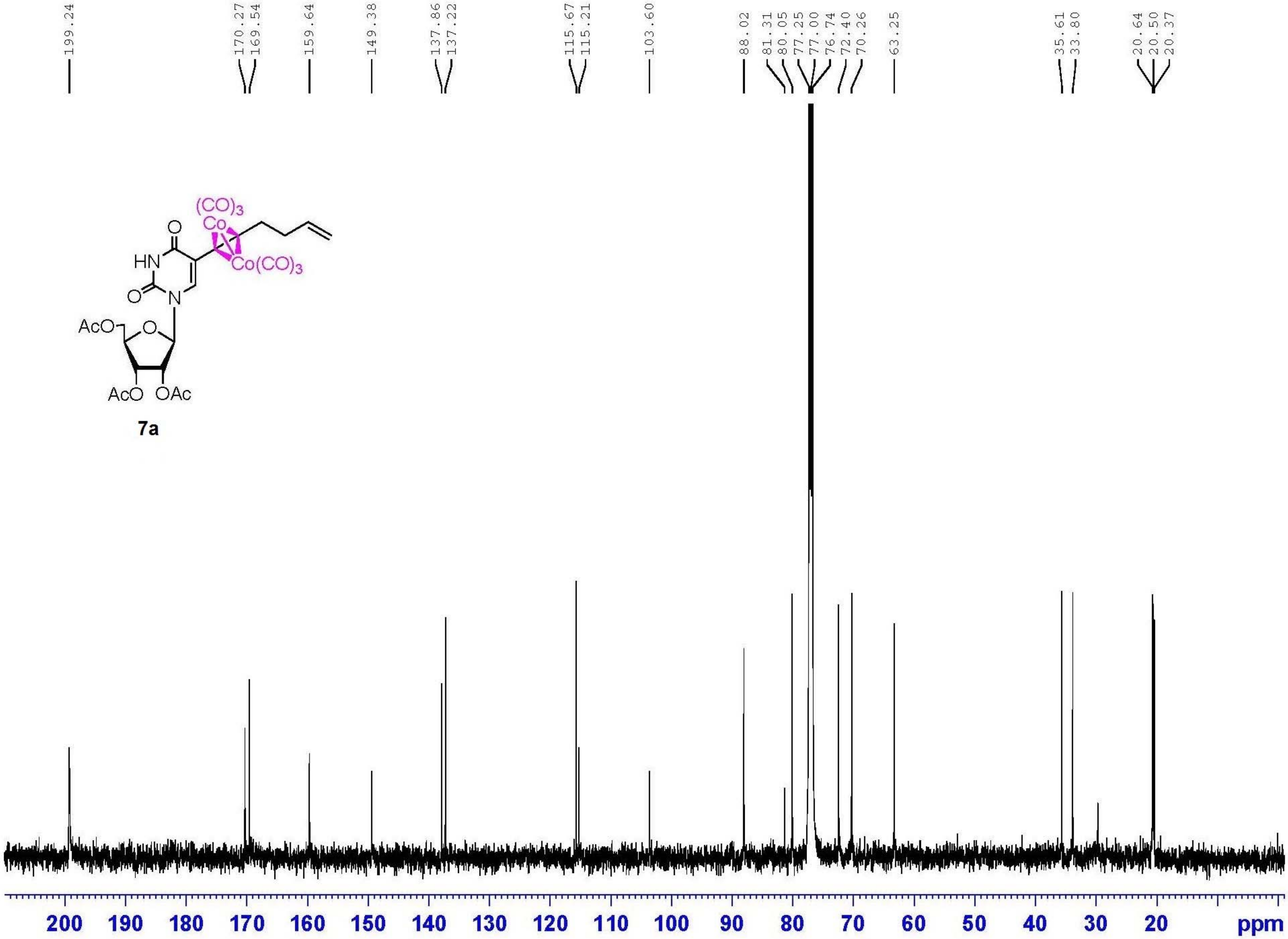

^{1}H NMR spectrum for **7b** ($CDCl_3$)

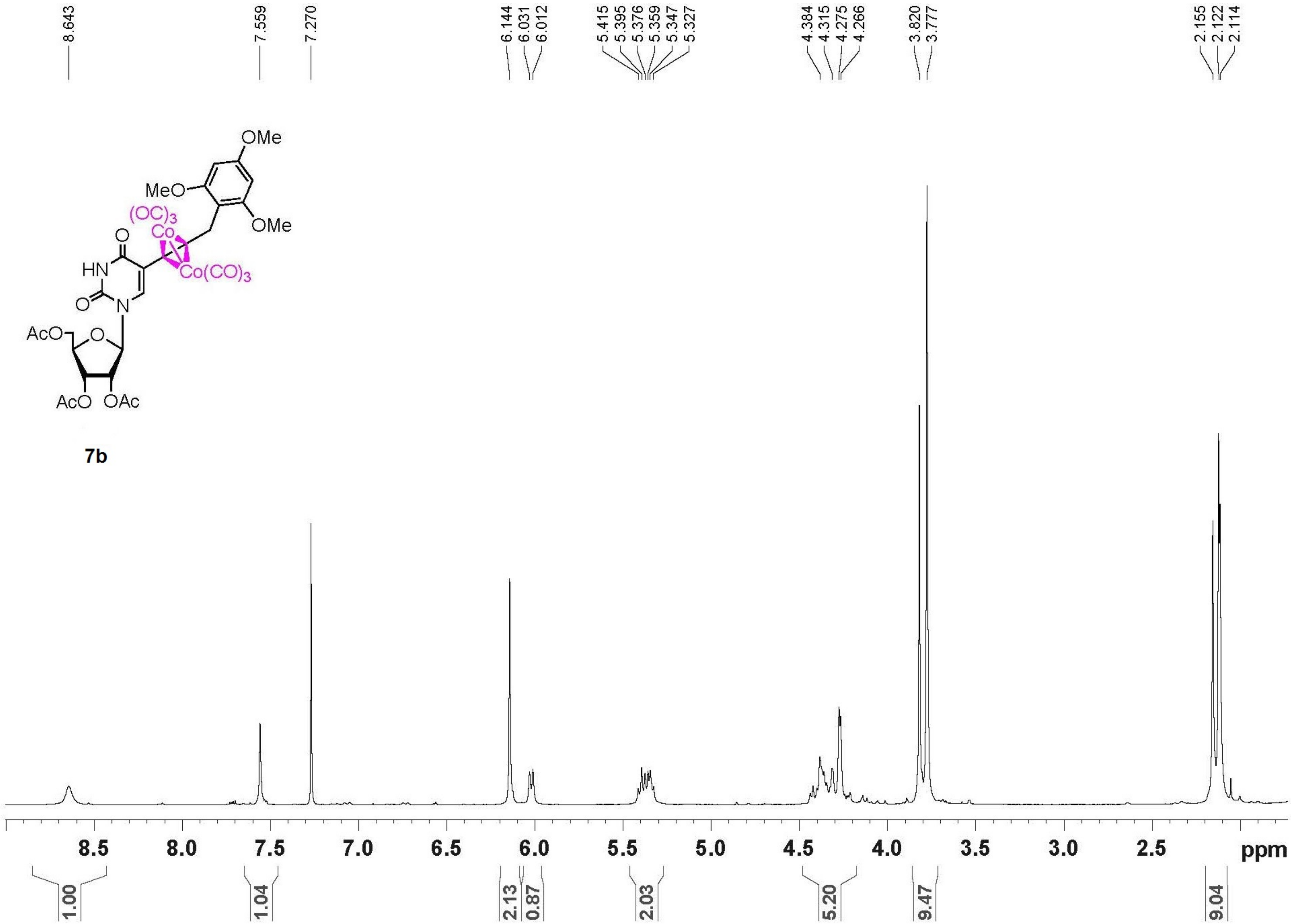

^{13}C NMR spectrum for **7b** ($CDCl_3$)

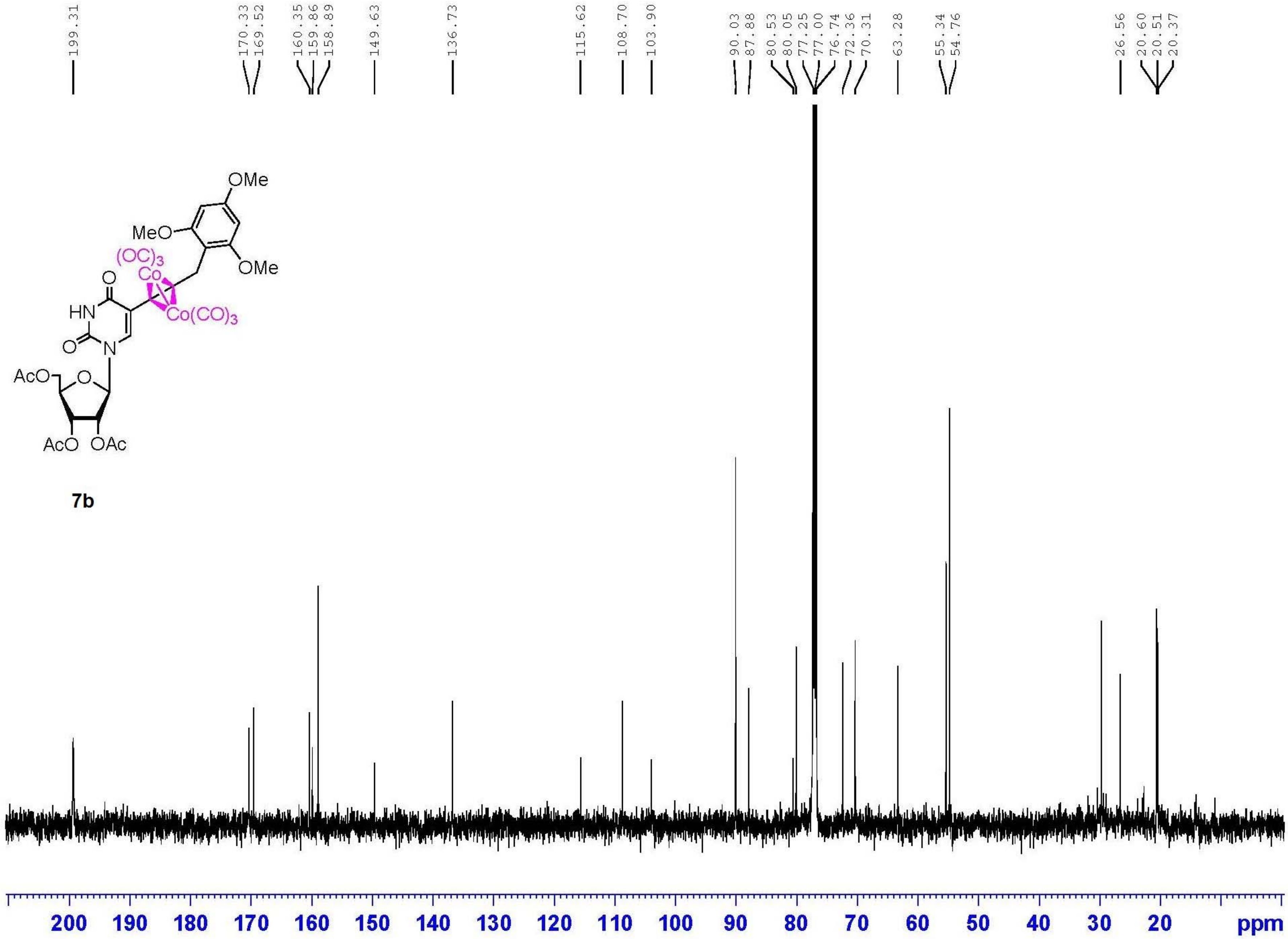

^{1}H NMR spectrum for **7d** ($CDCl_3$)

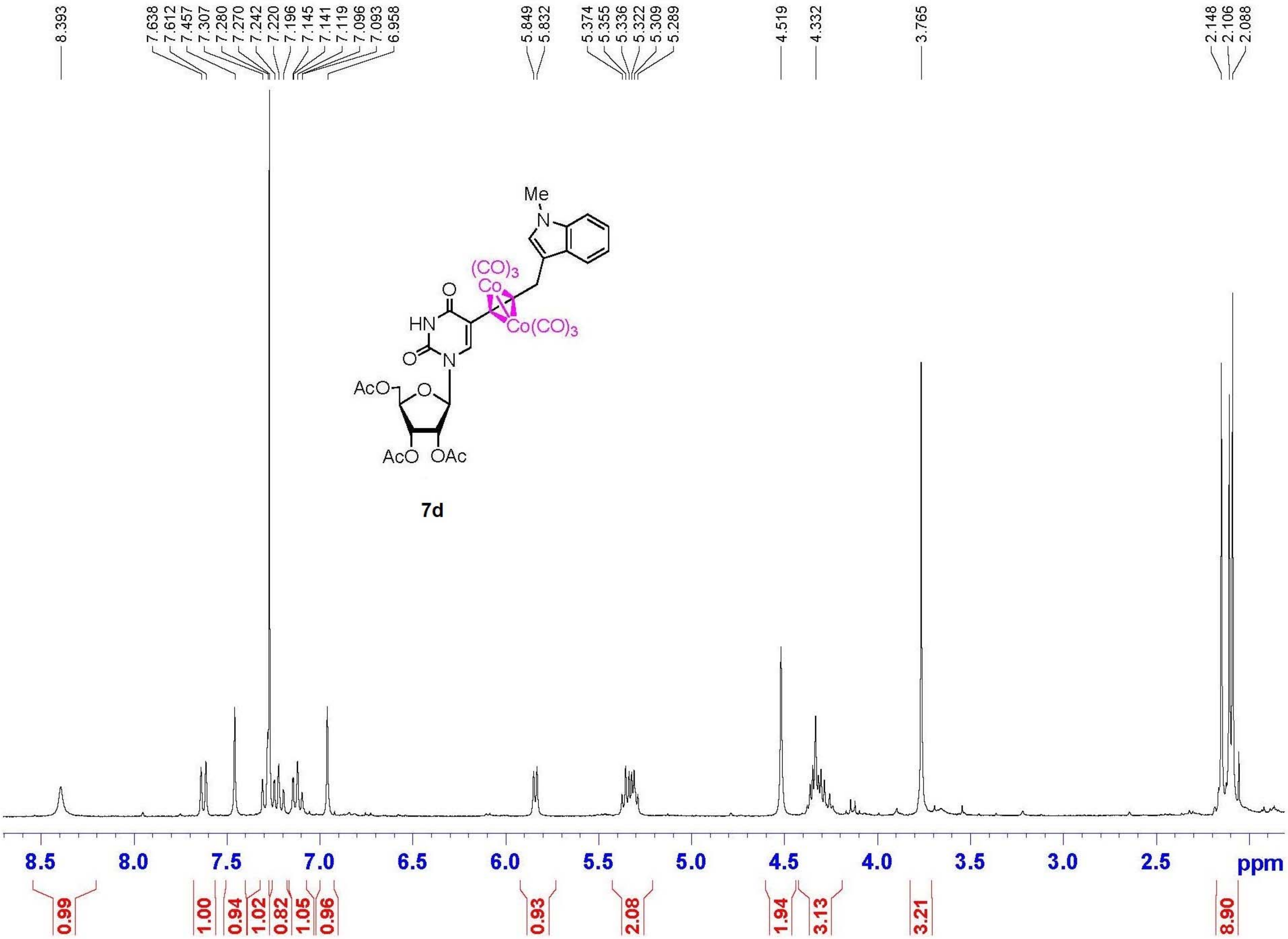

^{13}C NMR spectrum for **7d** ($CDCl_3$)

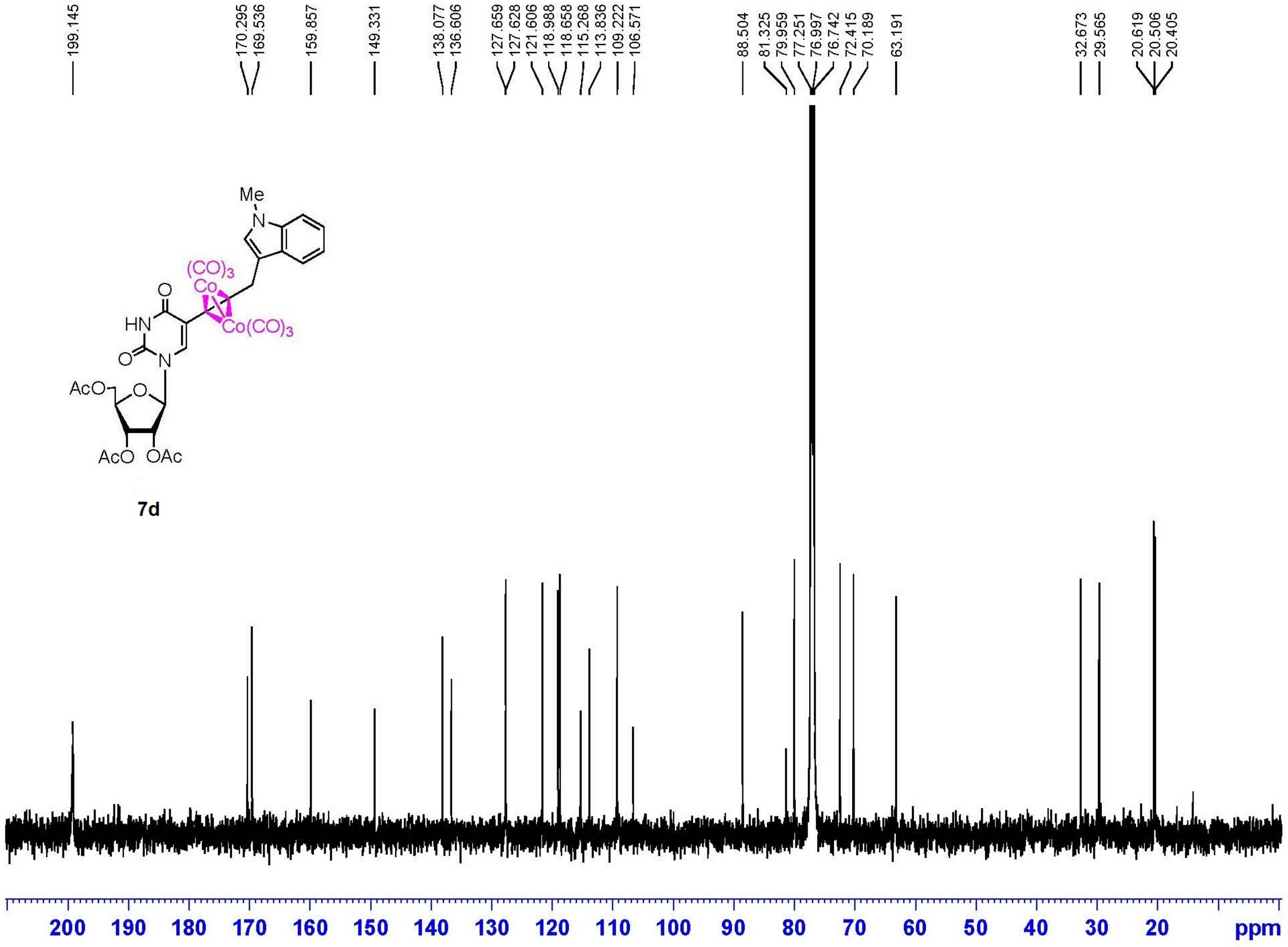

Supplement: File 1 — 1H and 13C NMR spectra for compounds 2, 3, 4, 5, 6a–c, and 7a,b,d. [file Beilstein_J_Org_Chem-16-01-s001.pdf]
